# Massive gene losses in Asian cultivated rice unveiled by comparative genome analysis

**DOI:** 10.1186/1471-2164-11-121

**Published:** 2010-02-19

**Authors:** Hiroaki Sakai, Takeshi Itoh

**Affiliations:** 1 Division of Genome and Biodiversity Research, National Institute of Agrobiological Sciences, 2-1-2 Kannondai, Tsukuba, Ibaraki 305-8602, Japan

## Abstract

**Background:**

Rice is one of the most important food crops in the world. With increasing world demand for food crops, there is an urgent need to develop new cultivars that have enhanced performance with regard to yield, disease resistance, and so on. Wild rice is expected to provide useful genetic resources that could improve the present cultivated species. However, the quantity and quality of these unexplored resources remain unclear. Recent accumulation of the genomic information of both cultivated and wild rice species allows for their comparison at the molecular level. Here, we compared the genome sequence of *Oryza sativa *ssp. *japonica *with sets of bacterial artificial chromosome end sequences (BESs) from two wild rice species, *O. rufipogon *and *O. nivara*, and an African rice species, *O. glaberrima*.

**Results:**

We found that about four to five percent of the BESs of the two wild rice species and about seven percent of the African rice could not be mapped to the *japonica *genome, suggesting that a substantial number of genes have been lost in the *japonica *rice lineage; however, their close relatives still possess their counterpart genes. We estimated that during evolution, *O. sativa *has lost at least one thousand genes that are still preserved in the genomes of the other species. In addition, our BLASTX searches against the non-redundant protein sequence database showed that disease resistance-related proteins were significantly overrepresented in the close relative-specific genomic portions. In total, 235 unmapped BESs of the three relatives matched 83 non-redundant proteins that contained a disease resistance protein domain, most of which corresponded to an NBS-LRR domain.

**Conclusion:**

We found that the *O. sativa *lineage appears to have recently experienced massive gene losses following divergence from its wild ancestor. Our results imply that the domestication process accelerated large-scale genomic deletions in the lineage of Asian cultivated rice and that the close relatives of cultivated rice have the potential to restore the lost traits.

## Background

With the worldwide demand for food crops increasing, the genetic improvement of cereals has been considered a promising approach to overcoming problems related to food supplies for the past several decades [1]. Between 1966 and 2000, the Green Revolution increased food production in densely populated developing countries by 125% [2]. The drastic improvement in crop yields has saved people in those countries from large-scale famine and economic upheaval [2]. However, the Food and Agriculture Organization (FAO) of the United Nations estimated that 854 million people worldwide remained undernourished from 2001 to 2003 http://www.fao.org/docrep/009/a0750e/a0750e00.HTM. Moreover, the United Nations Population Division has predicted that the world's population will increase from 6.5 billion in 2005 to 9.2 billion in 2050 http://www.un.org/esa/population/publications/wpp2006/wpp2006.htm. We will therefore need to produce 50% more grain supplies by 2025 [3].

Because crop yields have suffered serious losses due to various plant diseases, disease resistance has been one of the most important challenges in ensuring stable food supplies. It is estimated that diseases and insect pests can cause losses of up to 25% per year [4]. A further threat may be posed by global climate change [5], which may affect crop yields in various ways, such as increasing the risk of plant diseases or causing direct damage at specific developmental stages (e.g., flowering time) [6]. For these reasons, the development of new cultivars that show enhanced disease resistance, yield, and other aspects of performance is urgently needed. Wild relatives of cultivated species are expected to contain a wealth of genetic resources that are currently unexplored and may assist in the breeding of extant cultivars [1]. Despite the possible agronomic importance of such genetic resources, the amount and characteristics of these resources are largely unknown [7]. In this paper, we use a genome-wide comparative analysis to elucidate the quantity and agronomic potential of genes specific to wild rice species.

Asian cultivated rice, *Oryza sativa *L. (*Os*), contains two major cultivar groups, *japonica *(*Oj*) and *indica *(*Oi*), which are the most important grain crops and provide over 30% of the caloric intake in Asia http://www.irri.org/science/ricestat/index.asp. *Oj *and *Oi *split about 400,000 years ago [8], and were independently domesticated about 10,000 years ago [9]. The two cultivars show significant diversity in single nucleotide polymorphisms, intergenic sequences, and individual gene duplications, suggesting that the genomes of *Os *have undergone dynamic genome evolution [10]. In addition to *Os*, the genus *Oryza *includes the African cultivated rice, *O. glaberrima *(*Og*), as well as 22 wild species. The species have been classified into 10 genome types on the basis of chromosomal affinity during meiosis in experimental hybrids [11]. The *O. sativa *complex consists of *Os*, *Og*, and five wild species that have the same AA genome type. These species can be intercrossed, whereas different genome types are incompatible because of reproductive barriers [12]. Thus, two wild rice species of this complex, the annual *O. nivara *(*On*) and the perennial *O. rufipogon *(*Or*), which are considered the progenitors of *Os*, can be utilised in hybridisation-based breeding to develop new cultivars with favourable traits [7]. The premise of such breeding efforts is that a considerable amount of useful genetic resources is preserved in wild species. During the domestication process, rice appears to have undergone severe bottlenecks, which can be attributed to the initial cultivation of limited numbers of individuals that possessed key desirable traits, such as reduced grain shattering and seed dormancy [1,7]. The subsequent modern breeding process, which has been facilitated by extensive artificial selection for desirable traits, has resulted in the rapid loss of genetic diversity that was originally present in the wild ancestral population [13]. Therefore, there may be a significant number of genes that were not essential to the initial cultivation and domestication process, but that might be related to agronomically useful traits.

The term "wild rice-specific genes" can be used in two distinct senses: loci and alleles. Whereas allelic variations among cultivars have been used in breeding to improve agronomically important traits [14-16], few novel loci have been utilised. The discovery of wild rice-specific loci of agronomic importance would be beneficial to the genetic improvement of modern cultivars. In order to examine this issue, the genome sequence of an Asian cultivar, Nipponbare, which was released by the International Rice Genome Sequencing Project and annotated by the Rice Annotation Project (RAP) [17,18], is compared with the genomes of its wild relatives. The Oryza Map Alignment Project has made available a large collection of bacterial artificial chromosome end sequences (BESs) of wild rice species [19]. Herein, we use *Or*, *On*, and *Og *BESs to identify lost genes in *Oj *and *Oi*. The dynamic evolutionary changes evident in the rice genomes are discussed.

## Results

### Genomic deletions in the genome of *Oryza sativa *L. ssp. *japonica*

**Figure 1:**
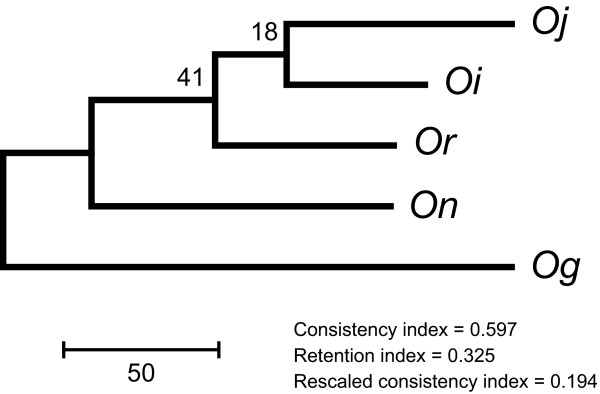
**Phylogenetic tree of the five *Oryza *species used in this study**: *Oj*, *O. sativa *L. ssp. *japonica*; *Oi*, *O. sativa *L. ssp. *indica*; *On*, *O. nivara*; *Or*, *O. rufipogon*; and *Og*, *O. glaberrima*. We employed the maximum-parsimony method using the third positions of 15,053 codons. Bootstrap values are shown above the internal branches. The scale indicates the branch length.

We mapped the BESs of two wild species, *On *and *Or*, and of the African cultivated rice species, *Og*, to the entire genome of *Oj*. The genome contigs of *Oi *were also aligned to the *Oj *genome. We selected 15,053 codons using the *Oj *genome annotation and constructed a phylogenetic tree of the five species using the maximum-parsimony (MP) method (Figure 1). Although a previous report revealed that *Oj *and *Oi *were derived independently from *Or *and *On*, respectively [20], the phylogenetic tree shows that the two Asian cultivated subspecies form the sister group of the wild rice species. We note, however, that the bootstrap values are quite low, as are the indices that measure the degree of homoplasy (Figure 1). We also reconstructed phylogenetic trees using the neighbour-joining (NJ) and the maximum-likelihood (ML) methods (Additional File 1). As expected, all the internal branches were not supported by the bootstrap test in both the NJ and ML trees. Several studies showed that the evolutionary relationship of the *Oryza *species is quite complicated and difficult to solve unambiguously [21-23]. That difficulty might be attributed to the lineage sorting [24]. Our results indicate that the phylogenetic relationships among Asian rice species cannot be uniquely determined by molecular sequence data, possibly because of incomplete speciation, variable gene histories due to the sorting of ancestral polymorphisms [25], or both.

The genomes of *Oryza *species vary in size. For example, *O. australiensis *has the largest genome, which is 960 Mb in size and is more than twice as large as the *Oj *genome [26]. A recent study has revealed that this variation in genome size is mainly due to the increase in retrotransposable elements in the *O. australiensis *lineage [26]. Thus, transposons may have caused the genome size differences observed between *Oj *and its relatives. We found that 38.9% of the *Oj *genome is composed of repetitive elements. The proportions of repetitive sequences in the BESs of *On*, *Or*, and *Og *are 39.7%, 39.1%, and 31.0%, respectively. Because BESs were fragmented DNA derived from genomes, these estimates may be biased. Therefore, to assess our BES analyses, we generated simulated BESs of *Oj *and *Oi*, and evaluated the amounts of repetitive sequences (see Methods, and Additional File 2). The proportions of the repetitive sequences in the simulated BESs of *Oj *(38.5%) and *Oi *(33.1%) were almost the same as those derived from the genome sequences (38.9% for *Oj *and 34.3% *Oi*), suggesting that the BESs of close relatives reflect the actual compositions of the repetitive sequences of the species used. The proportions of repeats in the two wild rice species are quite close to that in *Oj*, whereas *Og *possesses a relatively small fraction of repeats (Table 1). Because the estimated genome size of *Og *is about 20% smaller than those of the two wild rice species [27], the difference in the genome sizes can be explained in part by the differences in the amount of repetitive elements. By contrast, because *Oj*, *On*, and *Or *have nearly identical frequencies of repeats, the differences in the genome size between these species cannot be ascribed solely to increased or decreased repetitive elements. The lower repeat frequency of *Oi *than *Oj *may be because we did not use the unassembled reads and the unmapped pieces in which transposable elements were enriched [10]. Because the overall distribution of repetitive elements was similar among the examined species (Additional File 3), the differences in genome size do not seem to be due to expansion of a specific type of transposon.

**Table 1:** Statistical summary of the genomic sequences of four *Oryza *species used in this study.

	*Oj*	*On*	*Or*	*Og*
Genome size (Mbp)	382	448	439	354
No. of BESs	-	106,124	70,982	66,821
Total length (bp)	382,150,945	69,913,922	49,984,295	37,764,022
Total length of repetitive sequences (bp)	148,769,283	27,735,588	19,535,869	11,691,571
Fraction of repetitive sequences (%)	38.9	39.7	39.1	31.0
				
No. of BESs used for mapping	-	76,114	51,863	54,008
No. of mapped BESs	-	67,813	46,959	46,836
No. of ambiguous BESs	-	4,309	2,848	3,563
No. of unmapped BESs	-	3,992	2,056	3,609
Fraction of unmapped BESs (%)	-	5.2	4.0	6.7

*Oj*, *O. sativa *L. ssp. *japonica*; *On*, *O. nivara*; *Or*, *O. rufipogon*; and *Og*, *O. glaberrima*.

**Figure 2:**
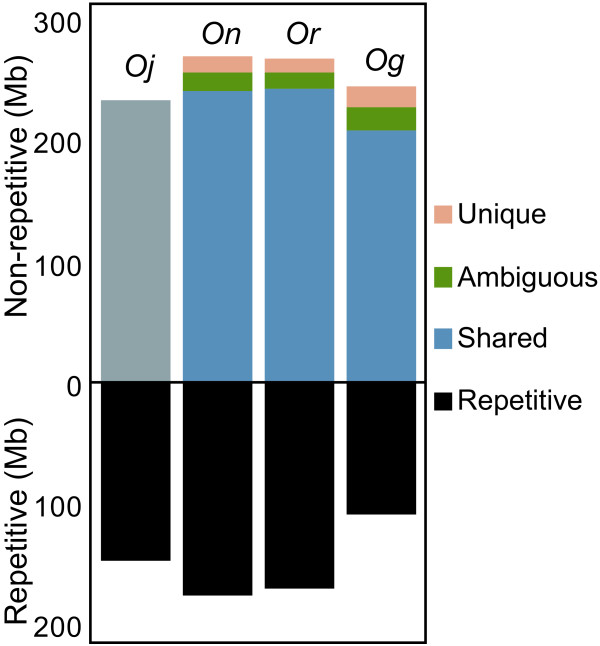
**Shared and unique genomic portions in four *Oryza *species**. Although the *Oj*-specific portion is unknown, the size of the shared region in *Oj *is expected to be nearly equal to that in *On *or *Or*.

If the genome size variation was caused by the accumulation of successive short insertions and deletions (indels), then the estimated insert lengths of the *Or *and *On *clones should be substantially greater than the interval sizes of paired BESs on the *Oj *genome. However, the average interval sizes of the two wild rice species on the *Oj *genome were almost the same as the insert length of the *Or *clones and were very close to that of the *On *clones (Additional File 4). In addition, although random indels in the two lineages should result in an equal amount of unique genomic regions in both species, the total genome lengths shared between *Oj *and its wild relatives (241, and 242 Mbp for *On*, and *Or*, respectively) were estimated to be nearly equal to the non-repetitive portion of the *Oj *genome (241 Mbp) (Figure 2). This finding indicates that only the wild rice genomes have retained a substantial quantity of unique regions, whereas the extant *Oj *genome lacks them (Figure 2). This observation suggests that only the *Oj *genome has undergone deletions that are larger in size than BAC clones, possibly during the domestication process. By contrast, the average interval size of *Og *was significantly greater than the estimated insert length (Additional File 4). If we take into consideration the fact that *Og *has fewer repeats than *Oj*, it appears that the difference in genome size between *Oj *and *Og *can be largely attributed to the successive accumulation of small-scale indels derived from repetitive sequences. Likewise, we estimated shared and unique genomic portions between *Oj *and *Oi*, using the mapping result of simulated BESs of *Oi *(Additional File 5).

Of the total BESs used for mapping, 10.9% and 9.5% of *On *and *Or *sequences, respectively, failed to be mapped to the *Oj *genome under the criteria of 80% identity and 70% coverage. Some BESs might have remained unmapped because of large alignment gaps that were derived from long DNA insertions into the *Oj *genome after the species split. However, even under the relaxed criteria of 80% identity and 30% coverage, 5.2% and 4.0% of the BESs of *On *and *Or*, respectively, could not be mapped (Table 1). Hence, our conservative estimate indicates that about 5% of the ancestral genome was lost in the *Oj *lineage after the divergence between *Oj *and its wild relatives. Likewise, 6.7% of the *Og *genome was missing in the *Oj *genome. Nonetheless, the unmapped BESs may have been conserved in the independent *Oi *lineage. In fact, of the 3,992, 2,056, and 3,609 unmapped BESs of *On*, *Or*, and *Og*, respectively, we found that 1,775, 1,047, and 880 BESs matched the *Oi *genome sequence under the criteria of 80% nucleotide identity and 30% sequence coverage. These results indicate that certain genome fractions were deleted only in the *Oj *lineage, following the divergence of the *japonica *and *indica *groups.

### Wild rice-specific gene content

The amount of DNA that is unique to the wild species can be estimated by counting the number of unmapped BESs. First, we estimated the total gene number (*n*_*g*_) as follows:

where *d *is the gene density of non-repetitive regions, *L *is the genome size, and *r *is the ratio of the non-repetitive regions to the total DNA. Given that the total number of *Oj *genes is ~32,000 [18], that the genome size is 382 Mbp, and that the ratio of non-repetitive DNA to total DNA is 0.61, *d *of *Oj *is 1.37 × 10^-4 ^per site. The gene densities of *On*, *Or*, and *Og *were estimated by counting the numbers of BESs that overlapped with protein-coding regions on the *Oj *genome, and then calculating the ratios of BESs of each species to simulated BESs of *Oj *(see Table 2, Methods, and Additional File 6). The genome sizes of *On*, *Or*, and *Og *were estimated to be 448, 439, and 354 Mbp [27], and the ratios of non-repetitive DNA to total DNA were 0.603, 0.609, and 0.690, respectively (Table 2). Therefore, the numbers of genes in the genomes of *On*, *Or*, and *Og *were estimated to be 42,356, 41,422, and 35,553, respectively (Table 2). We note here that the unmapped BESs contained fewer protein-coding genes than the mapped BESs. Natural selection against the loss of genes may reasonably account for this biased gene density. To take the biased gene distribution into account, we counted the number of BESs for which matches were found to sequence(s) in the non-redundant protein database (nr) of the National Center for Biotechnology Information. The ratio of the number (*n*_*u*_) of genes in the wild rice-specific regions to the number (*n*_*m*_) of genes in the shared regions should be equal to the ratio of the nr hits (*h*_*u*_) of unmapped BESs to the nr hits (*h*_*m*_) of mapped BESs, and therefore:

By substituting *n*_*m *_with *n*_*g *_- *n*_*u*_, we obtain

**Table 2:** Estimation of the numbers of species-specific genes.

	*On*		*Or*		*Og*		*Oj*	*Oi*
	*Oj*	*Oi*	*Oj*	*Oi*	*Oj*	*Oi*	*Oi*	*Oj*
*d*	1.57 × 10^-4^		1.55 × 10^-4^		1.45 × 10^-4^		1.37 × 10^-4^	1.32 × 10^-4^
*L *(Mbp)	448		439		354		382	466
*r*	0.603		0.609		0.690		0.611	0.666
*n*_*g*_	42,356		41,422		35,553		32,000	41,102
*n*_*u*_	1,360	1,105	934	865	1,260	1,456	946	980
*h*_*u*_	1,115	906	531	492	758	876	2,157	1,846
*h*_*m*_	33,614	33,823	23,016	23,055	20,631	20,513	70,794	75,588

*d*, gene density per site; *L*, genome size; *r*, fraction of non-repetitive DNA; *n*_*g*_, total number of genes; *n*_*u*_, number of genes missing in the genomes of *Oj *or *Oi*, but preserved in *On*, *Or*, or *Og*; *h*_*u*_, number of unmapped BESs that matched the nr database proteins; *h*_*m*_, number of mapped BESs that matched the nr database proteins. For *Oj *and *Oi*, we used simulated BESs.

We regarded "ambiguous" BESs (Table 1) as mapped BESs, so that the estimated numbers of unique genes would be conservative. As a result, we estimated that 1,360, 934, and 1,260 genes lost in *Oj *have been preserved in *On*, *Or*, and *Og*, respectively (Table 2). Likewise, the number of genes lost in the *Oi *genome was estimated (Table 2). In addition, to count all the possible candidates, we also estimated the numbers of unique genes, regarding the ambiguously mapped BES as unmapped ones (Additional File 7). Because the ratio of *h*_*u *_to *h*_*m *_depends on the criterion of similarity against the nr database proteins, we tested several criteria and obtained essentially the same results (Additional File 8).

Furthermore, we examined the portions of the genomes lost after the split of the *japonica *and *indica *cultivars. Using simulated BESs of *Oj *and *Oi*, we mapped the BESs to the other genomes, and estimated the number of unique genes in the *Oi *genome that are missing in the *Oj *genome, and vice versa (Table 2). We used 466 Mbp as the genome size of *Oi *as reported by a previous study [10]. It was revealed that *Oj *lost 980 genes, while *Oi *lost 946, which are comparable with the numbers for *On*, *Or*, and *Og*.

**Figure 3:**
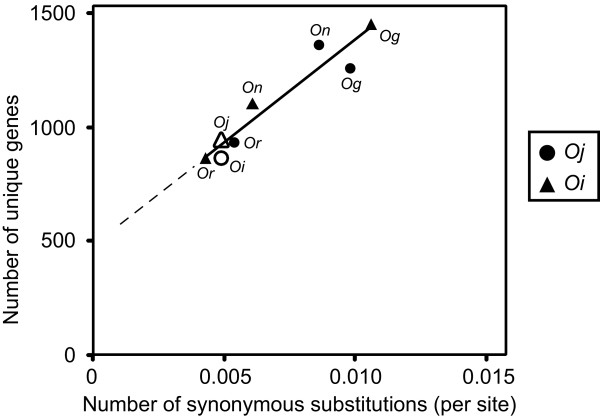
**Relationship between the numbers of synonymous substitutions and unique genes**. Black circles represent the numbers of unique genes that are missing from the *Oj *genome, and black triangles represent the numbers of unique genes that are missing from the *Oi *genome. A white circle represents the number of *Oi*-specific genes missing from the *Oj *genome, and a white triangle represents the number of *Oj*-specific genes missing from the *Oi *genome. The regression line indicates a constant rate of gene deletion over time.

If a gene loss occurs in a selectively neutral manner, the rate of the loss is constant, and the numbers of genes lost in *Oj *and *Oi *should increase proportionally with time. We assume that synonymous nucleotide substitutions are neutral, so that the number of substitutions corresponds to the elapsed time period. As expected, a linear relationship between the numbers of unique genes and synonymous substitutions was observed (Figure 3). If genes had been lost constantly throughout time, the regression line would cross the origin. However, Figure 3 indicates that the linear relationship is violated near the origin. This observation implies that the rate of gene loss has recently accelerated in the Asian cultivated rice lineage. It is possible that the rate of gene losses fluctuated during long-term evolution, and that the linear regression is not appropriate. However, it is rather difficult to imagine that genomic indel rates drastically changed during short-term evolution. Even if this was the case, our data clearly indicate that the rate accelerated after the split of wild and cultivated rice. The evolutionary distance between *Oi *and *Or *is 4.3 × 10^-3 ^substitutions per synonymous site. Therefore, given that the nucleotide substitution rate in rice is approximately 6.5 × 10^-9 ^per synonymous site per year [28], the acceleration seems to have occurred around 330,000 years ago or later.

### Functional biases of genes lost in the Asian cultivated rice lineage

**Figure 4:**
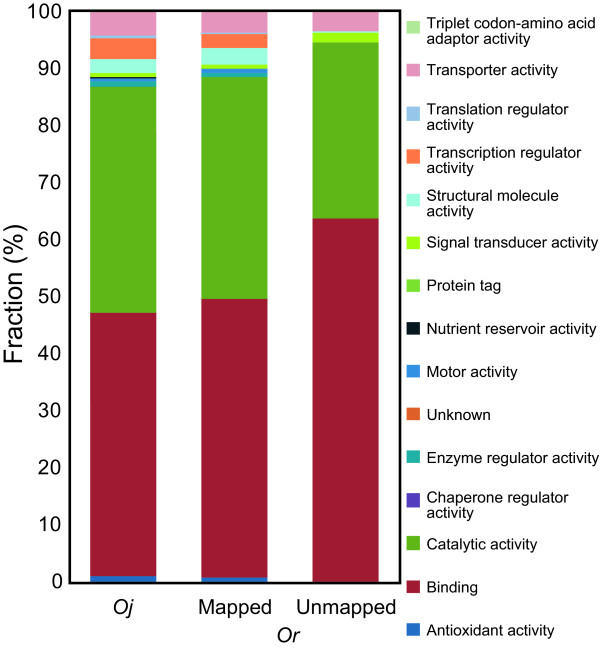
**Functional classifications of *Oj *and *Or *proteins**. The classifications of mapped and unmapped BESs of *Or *were derived from the nr database proteins that were homologous to the mapped and unmapped BESs (see Materials and Methods). Protein categories are based on the molecular functions of the Gene Ontology (GO) hierarchy.

Next, we turned our attention to the functional characteristics of the wild rice-specific gene regions. It was difficult to directly infer the functions of the wild rice-specific genes because the BESs were generally short, fragmented genomic sequences that did not cover a complete gene region. Therefore, we devised an indirect classification method in which the function of the most homologous protein in the nr database was used. Our BLASTX searches of unmapped BESs against the nr database revealed that 1,115 *On*, 531 *Or*, and 758 *Og *BESs had significant hits with an *E*-value of less than 10^-10 ^(Table 2). We also conducted BLASTN searches against full-length cDNAs of *Or *[29], but no significant hits were found. The functions of the BESs that encoded proteins were predicted by InterProScan against their homologues and subsequent Gene Ontology (GO) categorisation. We found that the overall pictures of the classifications of *Oj *genes and mapped BESs were nearly identical, and no statistically significant differences were observed (Figure 4 and Additional File 9). By contrast, among the 15 categories of molecular functions, genes with a "binding" function (GO:0005488) were overrepresented in the BESs unique to *On *(*p *= 1.0 × 10^-4^), *Or *(*p *= 1.1 × 10^-4^) and *Og *(*p *= 3.9 × 10^-3^). Similarly, when mapping all BESs to the *Oi *genome, we found that the *Oi *genome has lost a significant number of "binding"-related genes (Additional File 10). Functional classification of simulated BESs of *Oj *and *Oi *resulted in similar compositions. This result implies that the two subspecies of *Os *possess a certain number of binding-related genes that are missing in either genome (Additional File 11).

In order to investigate the detailed functions of these "binding" proteins, we examined the individual InterPro domains observed. We found that similar functions were listed for the 10 most frequently detected domains among the three species (Table 3 and Additional File 12). These domains may have appeared on the list by chance, because a large gene family has more opportunities to be deleted if deletion events occur randomly. Therefore, the excess of functional categories in the *On*-, *Or*- and *Og*-specific proteins was validated by Fisher's exact test. We found that three domains, leucine-rich repeat (IPR001611), NB-ARC (nucleotide-binding adaptor shared by apoptotic protease activating factor-1, R proteins, and *Caenorhabditis elegans *cell death gene 4) (IPR002182), and N-terminal leucine-rich repeat (IPR013210), were significantly overrepresented in each of the three species (*p *< 10^-3^) (Table 3 and Additional File 12). It should be noteworthy that the genes with nucleotide-binding site (NBS) and/or leucine-rich repeat (LRR) domains are known to be related only to disease resistance [30]. Thus, our finding suggests that several types of gene were deleted in the lineage leading to Asian cultivated rice, whereas a substantial number of disease resistance genes were preserved in *On*, *Or*, and *Og*. In total, 235 unmapped BESs of the three species were matched to 83 nr database proteins with a disease resistance protein domain (IPR000767), most of which were the NBS-LRR type (Additional File 13).

**Table 3:** The ten most frequent domains among the unmapped BESs of *O. rufipogon*.

InterPro ID	Description	Mapped		Unmapped		*p *value
		No. of genes with the domain	No. of genes without the domain	No. of genes with the domain	No. of genes without the domain	
IPR000719	Protein kinase, core	731	4075	26	109	0.22
IPR001611	Leucine-rich repeat	441	4365	47	88	1.10 × 10^-5^
IPR001878	Zinc finger, CCHC-type	138	4668	12	123	7.30 × 10^-5^
IPR002182	NB-ARC	345	4461	37	98	2.61 × 10^-12^
IPR007527	Zinc finger, SWIM-type	63	4743	8	127	9.95 × 10^-4^
IPR008271	Serine/threonine protein kinase, active site	569	4237	22	113	0.14
IPR011009	Protein kinase-like	755	4051	29	106	0.07
IPR013210	Leucine-rich repeat, N-terminal	222	4584	21	114	1.93 × 10^-6^
IPR017441	Protein kinase ATP binding, conserved site	540	4266	21	114	0.13
IPR017442	Serine/threonine protein kinase-related	628	4178	24	111	0.12

For each domain, the numbers of genes with or without the domain are listed for mapped and unmapped BESs. *P *values were calculated by using Fisher's exact test.

### Phylogenetic analysis of wild rice-specific genes

**Figure 5:**
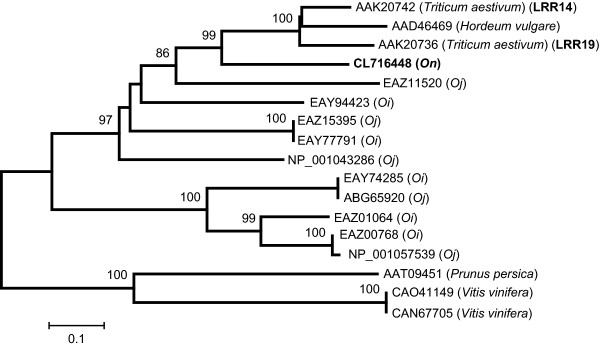
**Phylogenetic tree of possible disease resistance proteins**. CL716448 is a newly found homologue of *O. nivara *(*On*). Accession numbers and species names are shown. *Oj *stands for *O. sativa *L. ssp. *japonica *and *Oi *for *indica*. The tree was reconstructed by the neighbour-joining method [59]. The interior branches were tested by 1,000 bootstrap replicates, and bootstrap values of 50% or more are shown above the branches. The scale indicates the branch length.

Molecular phylogenetic trees are expected to show clear-cut evidence of gene losses in a large gene family. Figure 5 shows an example of a phylogenetic tree that includes protein sequences encoded in an *On*-specific DNA fragment, CL716448. This BES is missing in the *Oj *genome, but is homologous to a hexaploid wheat protein, LRR14, which contains an NBS-LRR domain [31] with high amino acid identity (62%). The amino acid alignment showed that CL716448 also contains part of the leucine-rich repeat domain of LRR14 (Additional File 14). Another hexaploid wheat protein (LRR19) and a barley homologue (AAD46469) were placed near LRR14 in the phylogenetic tree (Figure 5). If an orthologue had been retained in the *japonica *lineage, there would be an *Oj *sequence next to CL716448 in the phylogenetic tree. However, because there are no homologues in *Os*, it appears that the orthologue to CL716448 was lost from the *Os *lineage after the species split.

To obtain a conservative estimate of the number of wild rice-specific genes, we applied a relaxed threshold to map BESs onto the *Oj *genome. Therefore, there may be other candidate genes that were erroneously mapped. These might be detected by careful checking of their molecular phylogeny. In fact, a BES from *On*, CL619881, was mapped to the *Oj *genome and matched an *Oj *gene, BAD33147, with 84% amino acid identity. However, the large evolutionary distance of 0.145 between CL619881 and BAD33147 clearly indicates that BAD33147 is not an orthologue of CL619881 (Additional File 15). Thus, a reasonable evolutionary scenario is that BAD33147 was duplicated before divergence of the *On *and *Oj *lineages, and the *Oj *orthologue to CL619881 was subsequently lost. Because CL619881 is found in a group of genes related to *Pib*, which is known to be an effective disease resistance gene against rice blast, this *On *gene might encode an agronomically useful trait. If we apply more stringent criteria to map BESs onto the *Oj *genome, more wild rice-specific genes that have the potential to genetically improve modern cultivated rice may be identified.

## Discussion

### Drastic genome-wide deletions in Asian cultivated rice

Although grass genomes have been suggested to be evolutionarily stable [32], genome-wide studies in a variety of species, including rice, have shown dynamic evolution of their genome structures [10,33,34]. Our comparative analyses between cultivated and wild rice species revealed that Asian cultivated rice species have accumulated genomic deletions since their divergence from their wild ancestors. However, the deleted genes may have been preserved in other closely related species. In fact, we found that the genomes of wild rice species harbour about one thousand unique genes, which account for 3% of the total genes of the ancestor of *Oj*. These estimated numbers of the unique genes depended on the genome sizes based on the flow cytometry [27]. Although flow cytometry was later found to have overestimated the genome size of *Oj*, the difference was quite small (1.29%) [17]. This indicates that our estimates were not affected largely by possible overestimations of the genome sizes. Thus, it appears that the Asian cultivated rice lineage underwent these drastic changes over a relatively short period of time. Additionally, because large-scale deletions, rather than successive small-scale deletions, were found, the changes may have occurred in a relatively small number of steps.

The linear relationship between the number of lost genes and synonymous substitutions suggests selective neutrality of the deletion events (Figure 3). This hypothesis is consistent with our finding that the fraction of unmapped BESs that matched nr database proteins was less than that of mapped BESs (28% vs. 47% for *On*, and 26% vs. 46% for *Or*). Deletions of protein-coding regions were selected against, whereas non-coding regions that were functionally less important for the species were more likely to be lost from the genome. By contrast, the rate of recent gene losses seems to be accelerated in the *Os *lineage. This observation may be related to our finding that some functional domains are overrepresented among the deleted genes (Figure 4, Table 3, Additional File 9, and Additional File 12). In particular, NBS-LRR-type disease resistance genes seem to have been prone to elimination from the *Oj *genome. It is possible that these disease resistance genes were artificially overrepresented because of the biases in the collection of the BESs examined. However, the BESs are randomly distributed throughout the genome (Additional File 16). In addition, the functional classifications of the mapped BESs of the three close relatives were almost the same as the classification of the *Oj *genes (Figure 4 and Additional File 9), indicating that there were no obvious biases and that the current BES set represented the overall characteristics of the genes of the three close relatives.

The accelerated reductive evolution might be due in part to purifying selection against the disease resistance genes. It is intuitively expected that disease resistance genes should have been fully utilised in cultivated rice because of their agronomical importance. However, a study in *Arabidopsis thaliana *has shown that the fitness costs of resistance tend to decrease the presence of disease resistance genes [35]. During the domestication process, Asian cultivated rice may have had less contact with pathogens in the human-controlled environment. Thus, disease resistance genes may have imposed a cost on the fitness in the cultivated species.

A selectively neutral process of accelerated rice genome reduction may be possible if we consider gene deletions in large gene families, such as NBS-LRR. It is known that NBS-LRR genes were subjected to birth-and-death evolution, where some genes in a gene family were maintained in the genome, while others were deleted or become nonfunctional [36,37]. In fact, 32% of the NBS-LRR genes of *Oj *were shown to be nonfunctional [38]. Thus, one possible explanation is that rapid turnover following the high rate of nonfunctionalisation has led to the elimination of NBS-LRR genes in the *Oj *genome. Genetic recombination is one of the mechanisms that contribute to the evolution of NBS-LRR genes in plants [37,39]. Because NBS-LRR genes have frequently been amplified and, in many cases, are arrayed in tandem, a possible mechanism for the elimination of these genes is unequal crossing-over. In fact, Chin et al. reported that unequal crossing-over led to deletion of the lettuce disease resistance gene, *Dm3 *[40]. Another possible reason for the gene deletions in rice is that the mating system of rice has rapidly changed from outbreeding to inbreeding during domestication. This hypothesis is supported by the observation that *Dm3 *was eliminated in an inbred line [40]. Cytological and theoretical studies have demonstrated that self-fertilisation enhances the frequency of recombination [41,42]. Therefore, the increased self-fertilisation rate may have led to a high frequency of unequal crossing-over in the *Os *genome, resulting in the rapid loss of NBS-LRR genes. When we conducted BLASTN searches between the unmapped BESs of *On *and *Or*, the result showed that 22% of the *On *unmapped BESs and 33% of the *Or *unmapped BESs hit to each other. Furthermore, NBS or LRR domains were frequent among the hit BESs, suggesting that these genes were commonly preserved in the genomes of the wild rice species. It is generally accepted that the domestication of rice began approximately 10,000 years ago [9]. Although the loss of a great number of genes over such a short period may appear implausible, the fact that the loss of *Dm3 *in the lettuce genome occurred in merely four generations [40] suggests that drastic large-scale deletions may be ubiquitous in cultivated species.

### Wild rice germplasm as a reservoir of genes of agronomic importance

For the breeding of modern food crops, in addition to extant cultivars, wild species are abundant genetic resources that have been largely unexplored to date. Therefore, in this study, we focus on the quantity and quality of genes that have been lost from the genomes of Asian cultivated rice but are preserved in its relatives. Among the promising candidate genes that we have found are a significant number of possible novel disease resistance genes, such as homologues of *Pib *and *PibH8 *(Additional File 15), which are well-studied disease resistance genes against rice blast [43]. In this study, we have examined only three accessions of three species. We expect that there are many undiscovered useful genes and allelic variations in wild rice.

Because all three relatives analysed in this study have the same AA genome type as *Os*, the genes found to be unique to the genomes of the three relatives can be transferred to the *Os *genome by conventional hybridisation-based approaches. The recent development of introgression lines of elite cultivars and wild rice species has facilitated the efficient detection of quantitative trait loci associated with yield-related and other morphological or physiological traits [44,45]. Therefore, the introgression lines can be used to further examine the functions of the candidate genes identified in our analyses. As shown by the transfer of a bacterial blight resistance gene, *Xa1*, from *indica *to *japonica *[46], the transgenic approach may also be useful for examining the functions of the candidate genes and for conferring beneficial traits to the species of interest.

## Conclusion

Throughout this study, we have emphasized the importance of novel genetic resources in species that have not been examined in depth at the molecular level. We found that many potentially useful genes remain unexplored in the wild relatives of Asian cultivated rice. Hence, the complete genome sequencing of a wide variety of wild rice species should further expedite genomic breeding, as well as comprehensive analyses of gene function [27,47]. The high-throughput sequencing of rice species will unveil the retained genetic resources and will promote the development of new cultivated rice varieties.

## Methods

### Sequence data and alignment

We used genome assembly build 4 of *Oryza sativa *L. ssp. *japonica *(*Oj*), which was released by the International Rice Genome Sequencing Project [17]. From DDBJ release 70, we extracted the following numbers of bacterial artificial chromosome end sequences (BESs) generated by the Oryza Map Alignment Project [11,19]: 106,124 of *O. nivara *(*On*); 70,982 of *O. rufipogon *(*Or*); and 66,821 of *O. glaberrima *(*Og*). The length distributions of the BESs of each species are shown in Additional File 17. The genome of *O. sativa *ssp. *indica *(*Oi*) was downloaded from DDBJ under the accession numbers CM000126-CM000137. Repetitive regions were masked in lower case using RepeatMasker (A.F. Smit and P. Green, unpublished) with the MIPS Repeat Element Database (mips-REdat) [48]. All sequences having <30 bp of non-repetitive nucleotide sequence were excluded from our analyses. In order to estimate the amounts of undetected repeats, we conducted all-to-all BLASTN searches of the BESs in each species. When we applied the threshold of nucleotide identity ≥95% and aligned length ≥100 bp, about 3-6% of the BESs had three or more hits. We further conducted BLASTX searches for these possibly repetitive BESs against the nr database, and found a number of BESs that hit to members of multi-gene families such as kinases. Hence, the fractions of undetected repeats seemed to be smaller, possibly a few percents, so that they did not largely affect our results. The positions of the BESs in the genome were primarily determined by BLASTN searches [49]. We used thresholds of ≥80% identity and ≥70% coverage. To obtain conservative estimates of genes missing in Asian cultivated rice, we defined unmapped BESs as those with identity of <80% or coverage of <30%. BESs that satisfied either identity of ≥80% or coverage of ≥30%, and that did not satisfy the thresholds of ≥80% identity and ≥70% coverage were classified as "ambiguous." Regions conserved between *Oj *and the other species were estimated on the basis of the fractions of mapped BESs. The sizes (*R*_*C*_) of the conserved regions were calculated as follows:

where *L *is the genome size, *r *is the fraction of non-repetitive sequences, and *m *is the fraction of mapped BESs. The genome sizes of Kim, H. et al. were used [27]. Genome-wide alignments between *Oj *and *Oi *were constructed using BLASTZ with the options of "C = 0, H = 2000, Y = 3400, and T = 4" [50,51]. Some unmapped BESs hit to rice proteins in the nr database (see " Functional classification of genes encoded in BESs"). However, the numbers of the hits were very small (24, 19, and 24 for *On*, *Or*, and *Og*, respectively), and they were negligible in our estimations.

### Simulation study

We generated simulated BESs of *Oj *and *Oi *from the genome sequences by extracting the same numbers of sequence fragments as the total number (243,927) of BESs of three close relatives with the same lengths. To randomly select DNA fragments, we used the Mersenne Twister algorithm as the uniform pseudorandom number generator [52]. We selected fragments without any ambiguous nucleotides, such as N. To confirm the efficiency of our BES mapping, we aligned the simulated BESs of *Oj *to the *Oj *genome, and *Oi *to the *Oi *genome. In addition, we randomly introduced nucleotide mutations in the simulated BESs of *Oj *so that the number of substitutions became 0.025 that was equal to the average number of nucleotide differences between *Oj *and *Og*. As a result, we confirmed that 99.9% of the simulated BESs as well as artificially mutated BESs were successfully mapped (Additional File 2).

### Molecular evolutionary analyses

BESs that were mapped to multiple positions with the same identity and coverage were discarded from the dataset. If multiple BESs were mapped to the same genomic position, the BES with the highest nucleotide identity against *Oj *was selected. The four pairwise alignments between *Oj *and the other species were compared, and the regions that were covered by all five species were used. We made multiple alignments for these segments using Clustal W (ver. 1.83) [53]. The positions of the protein-coding regions were determined based on the genome annotation of *Oj*, which was released by the Rice Annotation Project Database (RAP-DB) [54]. We used the protein-coding regions to construct a phylogenetic tree. Genes containing one or more gaps or internal stop codons were excluded. The alignments were concatenated into one large multiple sequence alignment. MP and NJ trees were constructed using MEGA4 [55]. A ML tree was reconstructed using PAUP* 4.0b10 [56]. We used only the third positions of the codons.

In order to investigate the relationship between evolutionary distances and the numbers of close relative-specific genes, we calculated the numbers of synonymous substitutions in the five species by the Nei-Gojobori method [57].

### Estimation of gene densities

Weight factors of gene densities of each species other than *Oryza sativa *were estimated as follows:

where *N*_pc _is the number of BESs that overlap with RAP protein-coing regions more than 50 bp on the *Oj *genome, *N*_all _is the number of all BESs used for mapping, *J*_pc _is the number of simulated BESs of *Oj *that overlap with RAP protein-coding regions more than 50 bp, and *J*_all _is the number of all simulated BESs of *Oj*. The gene densities of non-*Oj *species were the *Oj *gene density multiplied by a weight factor.

### Functional classification of genes encoded in BESs

To infer the functions of genes encoded in mapped and unmapped BESs, we conducted BLASTX searches against the non-redundant protein sequence database (nr) using a threshold *E*-value of < 1.0 × 10^-10^. The best homologues of the BESs reported by BLASTX were used for indirect functional inference. Because the *Oj *genome is about 5% incomplete [17], there may be unmapped BESs that correspond to unsequenced portions of the genome. In fact, some unmapped BESs matched the rice proteins in the nr database with high identities. Therefore, we excluded those possibly mapped BESs from our analysis of gene functions. We chose threshold amino acid identities of 96.0%, 95.8%, and 94.5% for *On*, *Or*, and *Og *BESs, respectively, so that 5% of the homologous BESs of each species were discarded (Additional File 18). The detected proteins and the representative *Oj *proteins from the RAP-DB annotation were subjected to InterProScan searches [58]. On the basis of the Gene Ontology (GO) hierarchy, the functions were categorised by the map2slim program with generic GO slims. Proteins that were classified as transposable elements (GO:0003964 and GO:0004803) were excluded. The functional classifications of the proteins were compared between the *Oj *proteins and the mapped or unmapped BESs.

To verify the validity of this indirect method, we compared the direct and indirect functional classifications of the rice protein sets obtained from RAP-DB. Because the rice proteins themselves were included in the nr database, all self-hits were discarded. We confirmed that there was no significant difference between the classifications (Additional File 19). Therefore, the indirect functional inference should correctly reflect the actual function.

## Abbreviations

BES: bacterial artificial chromosome end sequence; NBS-LRR: nucleotide-binding site and/or leucine-rich repeat; RAP: Rice Annotation Project; MP: maximum-parsimony; NJ: neighbour-joining; ML: maximum-likelihood; BAC: bacterial artificial chromosome; nr: non-redundant; GO: Gene Ontology; NB-ARC: nucleotide-binding adaptor shared by apoptotic protease activating factor-1, R proteins, and *Caenorhabditis elegans *cell death gene 4; DDBJ: DNA Data Bank of Japan; MIPS: munich information center for protein sequences.

## Authors' contributions

HS performed the majority of the work and contributed to the writing of this paper. TI was largely responsible for the design of the study and the writing of this paper. Both authors read and approved the final manuscript.

## Supplementary Material

Additional file 1 **Phylogenetic tree of the five *Oryza *species used in this study: *Oj*, *O. sativa *L. ssp. *japonica*; *Oi*, *O. sativa *L. ssp. *indica*; *On*, *O. nivara*; *Or*, *O. rufipogon*; and *Og*, *O. glaberrima***. We used (A) the neighbour-joining and (B) the maximum-likelihood methods using the third positions of 15,053 codons. We used Kimura's two-parameter method for the neighbour-joining tree. Bootstrap values are shown above the internal branches. The scale indicates the branch length. Click here for file

Additional file 2 Statistics of simulated BESs of *O. sativa*: *Oj*, *O. sativa *L. ssp. *japonica*; *Oi*, *O*. *sativa *L. ssp. *indica*. Click here for file

Additional file 3 **Fraction of repetitive elements in the genomic sequences of five species: *Oj*, *O. sativa *L. ssp. *japonica*; *Oi*, *O. sativa *L. ssp. *indica*; *On*, *O. nivara*; *Or*, *O. rufipogon*; *Og*, *O. glaberrima***. The classification of repetitive elements was based on the MIPS Repeat Element Database. Click here for file

Additional file 4 Distributions of interval sizes of paired BESs along the *Oj *genome. Click here for file

Additional file 5 **Shared and unique genomic portions in *Oj *and *Oi***. Although the *Oj*-specific portion is unknown, the size of the shared region in *Oj *is expected to be nearly equal to that in *Oi*. Click here for file

Additional file 6 **Rates of BESs that overlap with RAP2 proteins of more than 50 bp**. Gene densities of *On*, *Or*, and *Og *were estimated by counting the numbers of BESs that overlapped with protein-coding regions on the *Oj *genome of more than 50 bp, and calculating the ratios of BESs of each species to simulated BESs of *Oj*. Click here for file

Additional file 7 **Estimation of the numbers of species-specific genes**. In contrast to Table 2, the ambiguous BESs were regarded as unmapped BESs. Click here for file

Additional file 8 **Estimates of unique genes in the genomes of close relatives under three different criteria: *hu*, the number of unmapped BESs that matched nr database proteins; *hm*, the number of mapped BESs that matched nr database proteins; *nu*, the number of genes in the close relative-specific regions**. Because the numbers of unique genes depend on the ratio of *hu *to *hm*, we examined two other thresholds for similarity searches against the nr database, an E-value of < 1.0 × 10^-20 ^and < 1.0 × 10^-50^, in addition to an E-value of < 1.0 × 10^-10^. Although the ratios slightly decreased with stringent thresholds, the numbers of unique genes did not drastically change, suggesting that the three close relatives possess ~1,000 unique genes that are missing from the genomes of *Oj *and *Oi*. Click here for file

Additional file 9 **Functional classifications of the proteins of *Oj *and two close relatives, *On *and *Og***. The classifications of mapped and unmapped BESs of *On *and *Og *were derived from the nr database proteins that were homologous to the mapped and unmapped BESs. Protein categories were based on the molecular functions of the Gene Ontology (GO) hierarchy. Click here for file

Additional file 10 **Functional classifications of the proteins of *Oj *and three close relatives, *On*, *Or*, and *Og***. All BESs were mapped to the genome of *O. sativa *L. ssp. *indica *(*Oi*). The classifications of mapped and unmapped BESs of the close relatives were derived from the nr database proteins that were homologous to the mapped and unmapped BESs. Protein categories were based on the molecular functions of the Gene Ontology (GO) hierarchy. Click here for file

Additional file 11 **Functional classifications of the proteins of *Oj *and of simulated BESs of *Oj *and *Oi***. Simulated BESs of *Oj *were mapped to the genome of *Oi*, and vice versa. The classifications of mapped and unmapped BESs were derived from nr database proteins that were homologous to the mapped and unmapped BESs. Protein categories were based on the molecular functions of the GO hierarchy. Click here for file

Additional file 12 **The ten most frequent domains among the unmapped BESs of *On *and *Og***. For each domain, the numbers of genes with or without the domain are listed for the mapped and unmapped BESs. *P *values were calculated using Fisher's exact test. Click here for file

Additional file 13 **List of 83 disease resistance-related genes that matched the unmapped BESs of *On*, *Or*, and *Og***. BESs that had internal stop codons within the aligned regions are presented in parentheses. Click here for file

Additional file 14 **Amino acid alignment between the *On*-specific disease resistance gene, CL716448, and its homologues**. Amino acid sequences with black backgrounds indicate leucine-rich repeat domains predicted by InterProScan searches. Click here for file

Additional file 15 **Phylogenetic tree of possible disease resistance proteins**. CL619881 is a newly found homologue from *On*. Accession numbers and species names are shown. The tree was reconstructed by the neighbour-joining method. The interior branches were tested by 1,000 bootstrap replicates, and bootstrap values of 50% or greater are shown above the branches. The scale indicates the branch length. Click here for file

Additional file 16 **Density of the BESs throughout the *Oj *genome**. Numbers of the BESs were counted by using a sliding window of 1 Mbp width with a step size of 500 Kbp. Click here for file

Additional file 17 Length distributions of BESs of three close relatives Click here for file

Additional file 18 Distributions of the amino acid identities of mapped BESs against the top-hit nr database proteins with a threshold of 10^-10^ Click here for file

Additional file 19 **Indirect functional classifications of the genes of *Oj***. We obtained protein-coding nucleotide sequences of representative sequences for each gene of *Oj *from RAP-DB and conducted BLASTX searches against the nr database with a threshold of 10^-10^. Indirect classifications were facilitated using four sets of the nr database proteins: Top-hit, top-hit genes; ID = 90%, nr database proteins with amino acid identities over 90%; ID = 80%, nr database proteins with amino acid identities over 80%; ID = 70%, nr database proteins with amino acid identities over 70%. As a comparison, the functional classification of the representative sequences of the *Oj *genes (Rep) is shown on the right. Click here for file
