# Supplementary material for: Massive gene losses in Asian cultivated rice unveiled by comparative genome analysis
Source: BMC Genomics. 2010 Feb 19;11:121. doi: 10.1186/1471-2164-11-121 (PMC2831846; doi:10.1186/1471-2164-11-121)

Additional Data File 1. Phylogenetic tree of the five *Oryza* species used in this study: *Oj*, *O. sativa* L. ssp. *japonica*; *Oi*, *O. sativa* L. ssp. *indica*; *On*, *O. nivara*; *Or*, *O. rufipogon*; and *Og*, *O. glaberrima*. We used (A) the neighbour-joining and (B) the maximum-likelihood methods using the third positions of 15,053 codons. We used Kimura' s two-parameter method for the neighbour-joining tree. Bootstrap values are shown above the internal branches. The scale indicates the branch length.

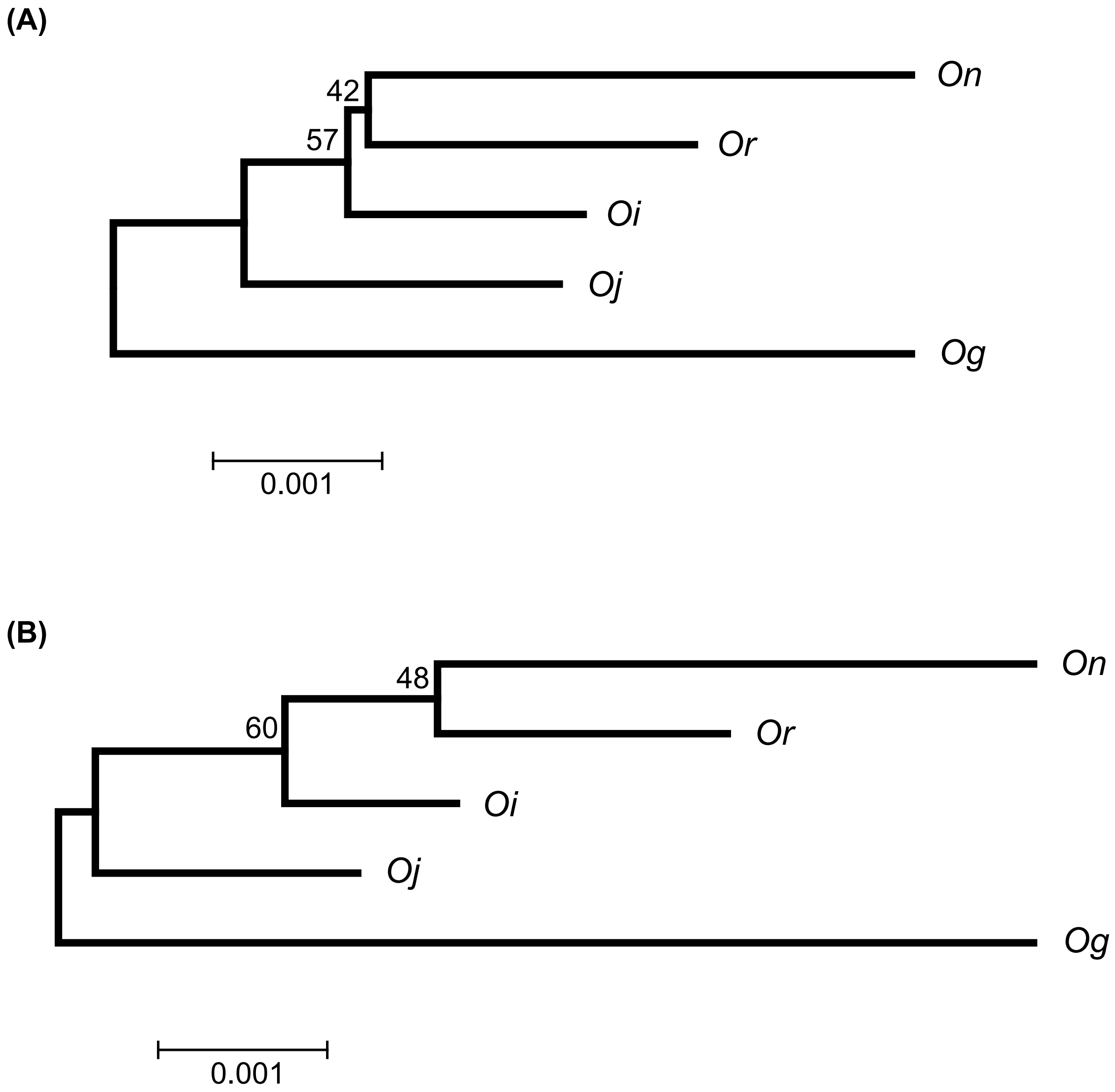

Supplement: Additional file 1 — Phylogenetic tree of the five Oryza species used in this study: Oj, O. sativa L. ssp. japonica; Oi, O. sativa L. ssp. indica; On, O. nivara; Or, O. rufipogon; and Og, O. glaberrima. We used (A) the neighbour-joining and (B) the maximum-likelihood methods using the third positions of 15,053 codons. We used Kimura's two-parameter method for the neighbour-joining tree. Bootstrap values are shown above the internal branches. The scale indicates the branch length. [file 1471-2164-11-121-S1.PDF]
