# Supplementary material for: Massive gene losses in Asian cultivated rice unveiled by comparative genome analysis
Source: BMC Genomics. 2010 Feb 19;11:121. doi: 10.1186/1471-2164-11-121 (PMC2831846; doi:10.1186/1471-2164-11-121)
Supplement: Additional file 2 — Statistics of simulated BESs of O. sativa: Oj, O. sativa L. ssp. japonica; Oi, O. sativa L. ssp. indica. [file 1471-2164-11-121-S2.PDF]

Additional Data File 2. Statistics of simulated BESs of *O. sativa*: *Oj*, *O. sativa* L. ssp. *japonica*; *Oi*, *O. sativa* L. ssp. *indica*.

		Oj	*Oi*
No. of simulated BESs		243,927	
Total nucleotide length (bp)		157,674,886	
Fraction of repetitive sequences (%)	Simulated BESs	38.5	33.1
	Genome	38.9	34.3
Fraction of mapped BESs (%)	*Oj* genome	99.9	88.1
	Oi genome	90.4	99.9
