# Supplementary material for: Massive gene losses in Asian cultivated rice unveiled by comparative genome analysis
Source: BMC Genomics. 2010 Feb 19;11:121. doi: 10.1186/1471-2164-11-121 (PMC2831846; doi:10.1186/1471-2164-11-121)

Additional Data File 3. Fraction of repetitive elements in the genomic sequences of five species: *Oj*, *O. sativa* L. ssp. *japonica*; *Oi*, *O. sativa* L. ssp. *indica*; *On, O. nivara*; *Or, O. rufipogon*; *Og, O. glaberrima*. The classification of repetitive elements was based on the MIPS Repeat Element Database.

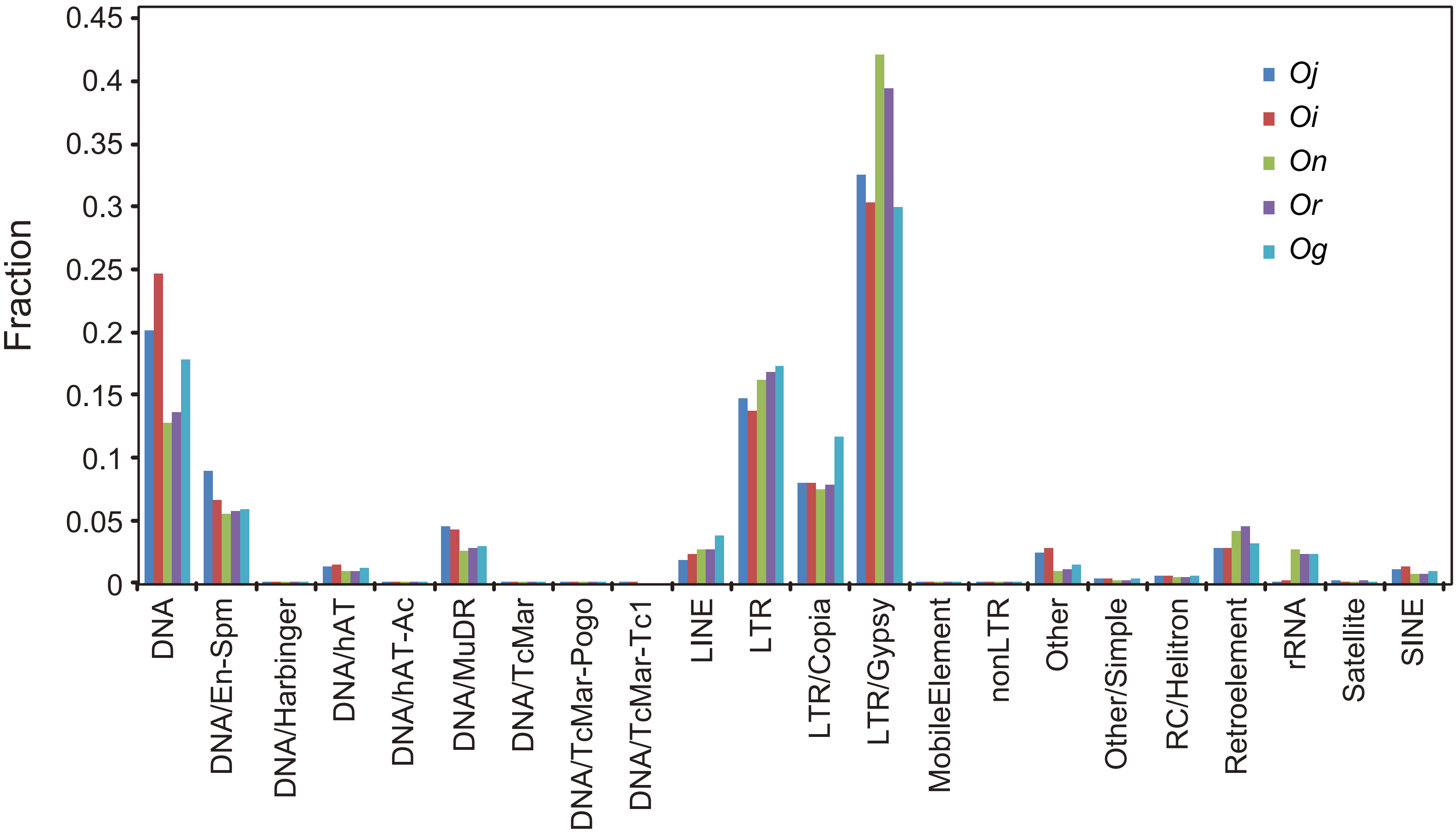

Supplement: Additional file 3 — Fraction of repetitive elements in the genomic sequences of five species: Oj, O. sativa L. ssp. japonica; Oi, O. sativa L. ssp. indica; On, O. nivara; Or, O. rufipogon; Og, O. glaberrima. The classification of repetitive elements was based on the MIPS Repeat Element Database. [file 1471-2164-11-121-S3.PDF]
