# Supplementary figures and images for: Massive gene losses in Asian cultivated rice unveiled by comparative genome analysis
Source: BMC Genomics. 2010 Feb 19;11:121. doi: 10.1186/1471-2164-11-121 (PMC2831846; doi:10.1186/1471-2164-11-121)

**Additional Data File 4.** Distributions of interval sizes of paired BESs along the *Oj* genome.

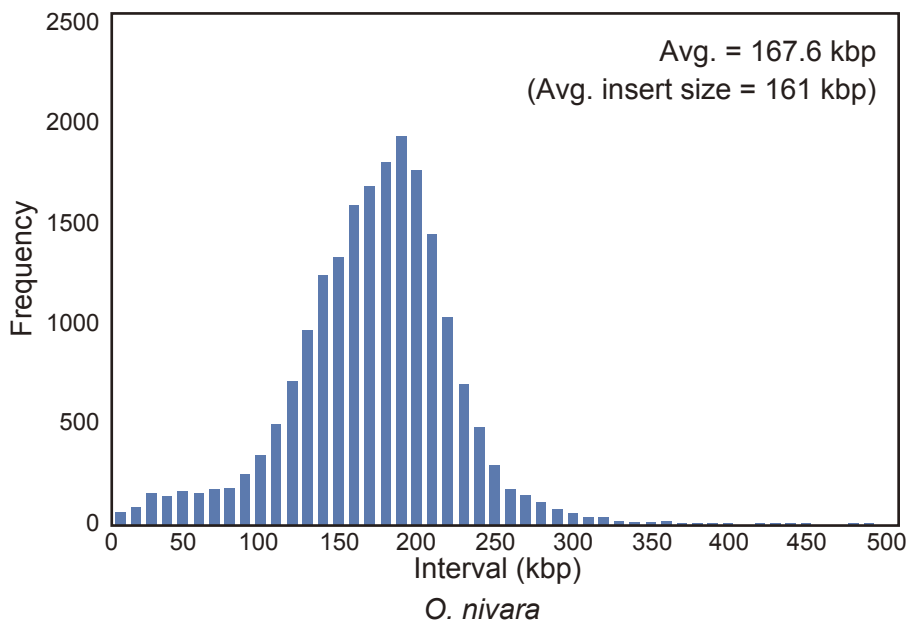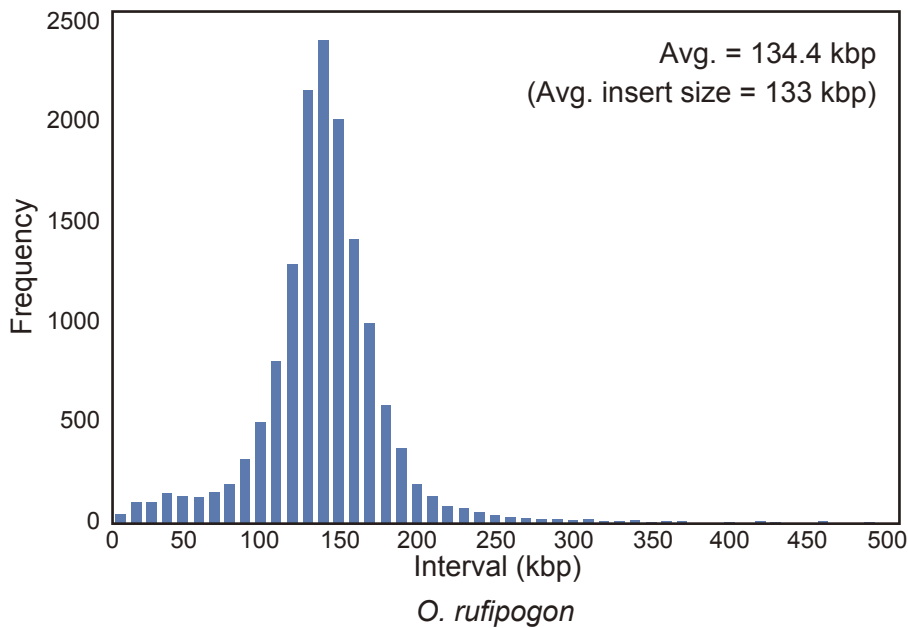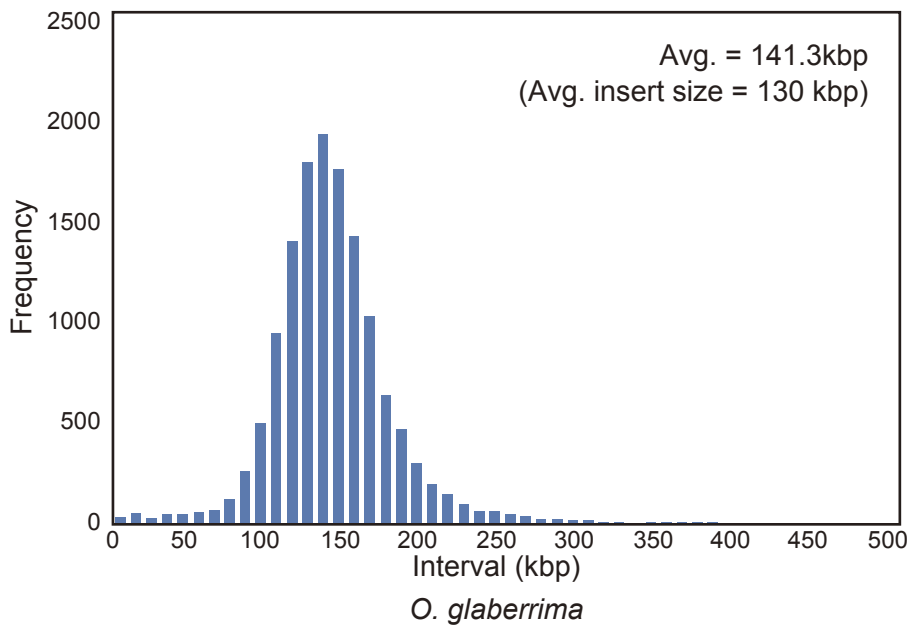

Supplement: Additional file 4 — Distributions of interval sizes of paired BESs along the Oj genome. [file 1471-2164-11-121-S4.PDF]

**Additional Data File 17.** Length distributions of BESs of three close relatives.

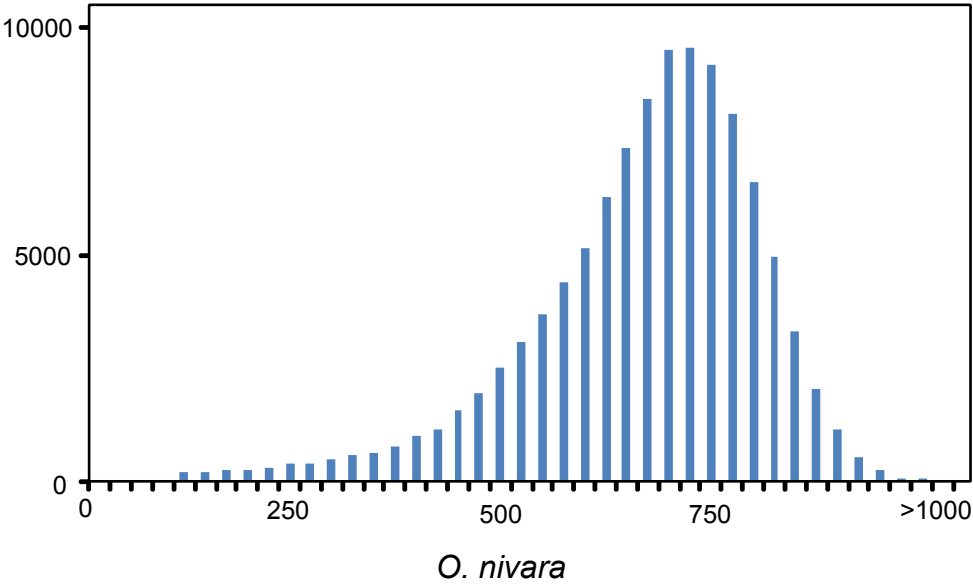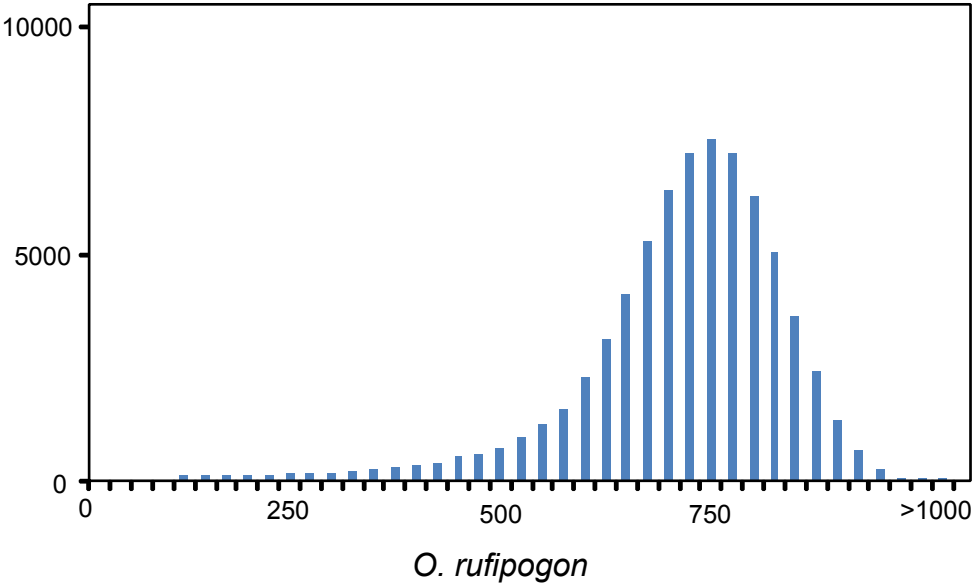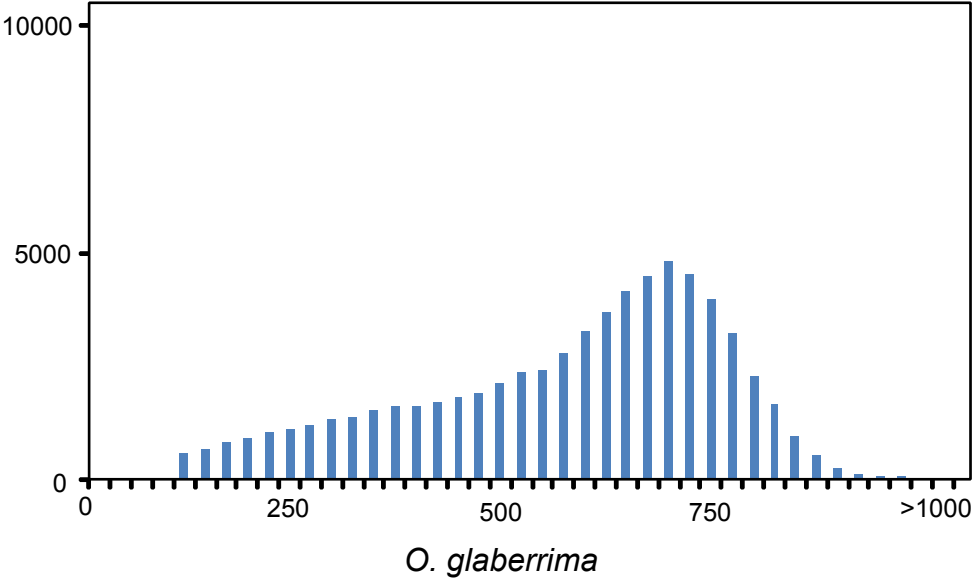

Supplement: Additional file 17 — Length distributions of BESs of three close relatives [file 1471-2164-11-121-S17.PDF]
