# Supplementary material for: Massive gene losses in Asian cultivated rice unveiled by comparative genome analysis
Source: BMC Genomics. 2010 Feb 19;11:121. doi: 10.1186/1471-2164-11-121 (PMC2831846; doi:10.1186/1471-2164-11-121)

Additional Data File 5. Shared and unique genomic portions in *Oj* and *Oi*. Although the *Oj*-specific portion is unknown, its size is expected to be nearly equal to that of *Oi*.

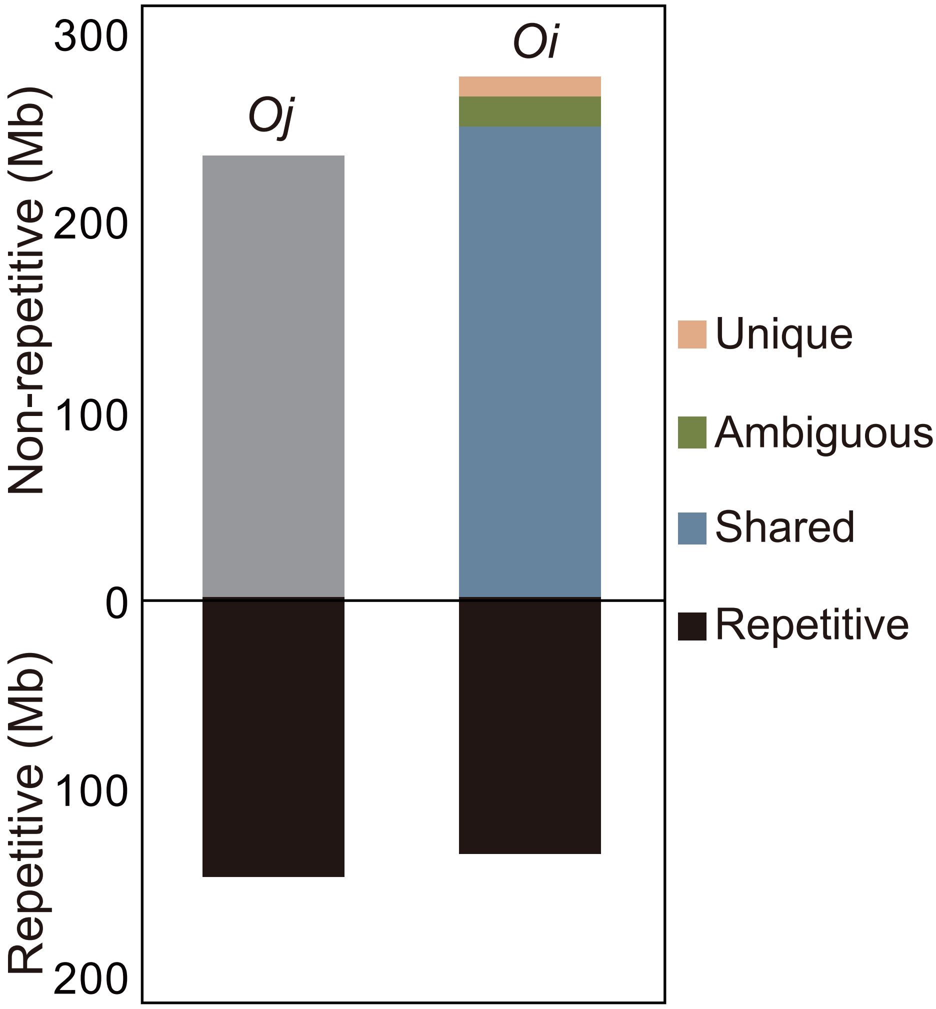

Supplement: Additional file 5 — Shared and unique genomic portions in Oj and Oi. Although the Oj-specific portion is unknown, the size of the shared region in Oj is expected to be nearly equal to that in Oi. [file 1471-2164-11-121-S5.PDF]
