# Supplementary material for: Massive gene losses in Asian cultivated rice unveiled by comparative genome analysis
Source: BMC Genomics. 2010 Feb 19;11:121. doi: 10.1186/1471-2164-11-121 (PMC2831846; doi:10.1186/1471-2164-11-121)
Supplement: Additional file 6 — Rates of BESs that overlap with RAP2 proteins of more than 50 bp. Gene densities of On, Or, and Og were estimated by counting the numbers of BESs that overlapped with protein-coding regions on the Oj genome of more than 50 bp, and calculating the ratios of BESs of each species to simulated BESs of Oj. [file 1471-2164-11-121-S6.PDF]

Additional data file 6. Rates of BESs that overlap with RAP2 proteins of more than 50 bp.

	Oj	*Oi*	*Or*	*On*	*Og*
No. of mapped BESs	187,419	201,209	51,863	76,114	54,008
No. of mapped BESs that overlap with more than 50 bp of RAP protein-coding regions	21,794 (11.6%)	22,610 (11.2%)	6,818 (13.1%)	10,126 (13.3%)	6,670 (12.4%)
Ratio to *Oj*	1.00	0.97	1.13	1.15	1.07

NOTE. - Gene densities of *On*, *Or*, and *Og* were estimated by counting the numbers of BESs that overlapped with over 50 bp of protein-coding regions of the *Oj* genome of more than 50bp, and calculating the ratios of BESs of each species to simulated BESs of *Oj*.
