# Supplementary material for: Massive gene losses in Asian cultivated rice unveiled by comparative genome analysis
Source: BMC Genomics. 2010 Feb 19;11:121. doi: 10.1186/1471-2164-11-121 (PMC2831846; doi:10.1186/1471-2164-11-121)
Supplement: Additional file 7 — Estimation of the numbers of species-specific genes. In contrast to Table 2, the ambiguous BESs were regarded as unmapped BESs. [file 1471-2164-11-121-S7.PDF]

Additional Data File 7. **Estimation of the numbers of species-specific genes.** *d*, gene density per site; *L*, genome size; *r*, fraction of non-repetitive DNA; n_g, total number of genes; n_u, number of genes missing in the genomes of *Oj* or *Oi*, but preserved in *On*, *Or*, or *Og*; h_u, number of unmapped BESs that matched the nr database proteins; h_m, number of mapped BESs that matched the nr database proteins. In contrast to Table 2, the ambiguous BESs were regarded as unmapped BESs. For *Oj* and *Oi*, we used simulated BESs.

	On		*Or*		*Og*		*Oj*	*Oi*
	Oj	*Oi*	*Oj*	*Oi*	*Oj*	*Oi*	*Oi*	*Oj*
d	1.57×10^{-4}		1.55×10^{-4}		1.45×10^{-4}		1.37×10^{-4}	1.32×10^{-4}
L (Mbp)	448		439		354		382	466
r	0.603		0.609		0.690		0.611	0.666
n_g	42,356		41,422		35,553		32,000	41,129
n_u	2,891	2,332	2,449	2,287	2,779	3,148	2,843	2,657
h_u	2,370	1,912	1,392	1,300	1,672	1,894	6,585	5,039
h_m	32,359	32,817	22,155	22,247	19,717	19,495	67,546	72,973
