# Supplementary material for: Massive gene losses in Asian cultivated rice unveiled by comparative genome analysis
Source: BMC Genomics. 2010 Feb 19;11:121. doi: 10.1186/1471-2164-11-121 (PMC2831846; doi:10.1186/1471-2164-11-121)
Supplement: Additional file 8 — Estimates of unique genes in the genomes of close relatives under three different criteria: hu, the number of unmapped BESs that matched nr database proteins; hm, the number of mapped BESs that matched nr database proteins; nu, the number of genes in the close relative-specific regions. Because the numbers of unique genes depend on the ratio of hu to hm, we examined two other thresholds for similarity searches against the nr database, an E-value of < 1.0 × 10^-20^ and < 1.0 × 10^-50^, in addition to an E-value of < 1.0 × 10^-10^. Although the ratios slightly decreased with stringent thresholds, the numbers of unique genes did not drastically change, suggesting that the three close relatives possess ~1,000 unique genes that are missing from the genomes of Oj and Oi. [file 1471-2164-11-121-S8.PDF]

Additional Data File 8. Estimates of unique genes in the genomes of close relatives under three different criteria: h_u, the number of unmapped BESs that matched nr proteins; h_m, the number of mapped BESs that matched nr proteins; n_u, the number of genes in the close relatives-specific regions. Because the numbers of unique genes depend on the ratio of h_u to h_m, we examined two other thresholds for similarity searches against the nr database, *E*-value of < 1.0 x 10^{-20} and < 1.0 x 10^{-50}, in addition to an *E*-value of < 1.0 x 10^{-10}. Although the ratios slightly decreased with stringent thresholds, the numbers of unique genes did not drastically change, suggesting that the three close relatives possess ~ 1,000 unique genes that are missing from the genomes of *Oj* and *Oi*.

		h_u / h_m			n_u		
E-value		1.0 X 10^{-10}	1.0 X 10^{-20}	1.0 X 10^{-50}	1.0 X 10^{-10}	1.0 X 10^{-20}	1.0 X 10^{-50}
	On	0.0332	0.0313	0.0302	1,360	1,287	1,243
Oj	*Or*	0.0231	0.0216	0.0188	934	877	766
	Og	0.0367	0.0361	0.0298	1,260	1,239	1,028
	On	0.0268	0.0247	0.0227	1,105	1,022	942
Oi	*Or*	0.0213	0.0194	0.0182	865	789	741
	Og	0.0427	0.0418	0.0364	1,456	1,425	1,248
