# Supplementary material for: Massive gene losses in Asian cultivated rice unveiled by comparative genome analysis
Source: BMC Genomics. 2010 Feb 19;11:121. doi: 10.1186/1471-2164-11-121 (PMC2831846; doi:10.1186/1471-2164-11-121)

Additional Data File 9. Functional classifications of the proteins of *Oj* and two close relatives, *On* and *Og*. The classifications of mapped and unmapped BESs of *On* and *Og* were derived from nr proteins that were homologous to the mapped and unmapped BESs (see Methods in the main text). Protein categories were based on the molecular functions of the Gene Ontology (GO) hierarchy.

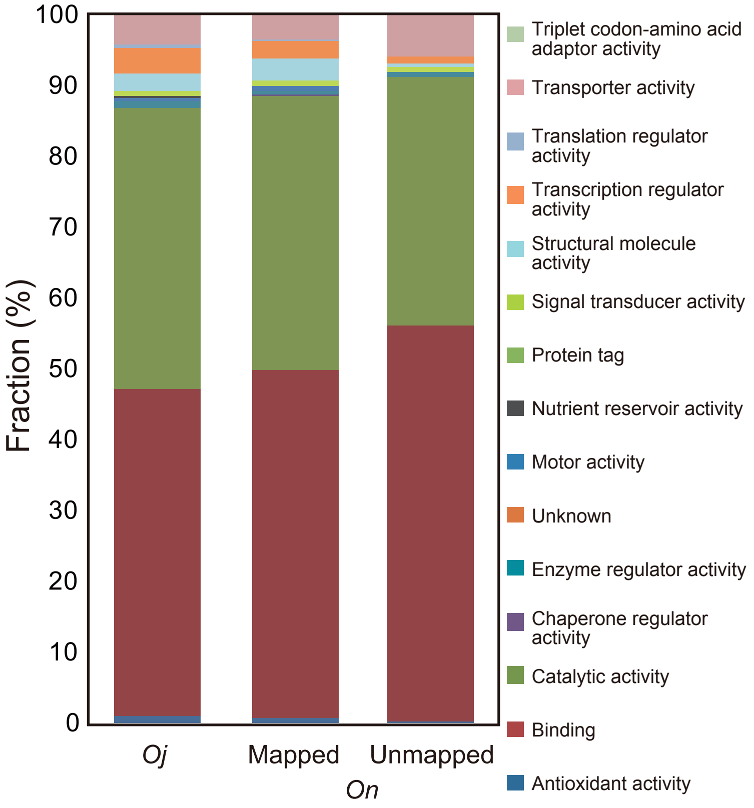

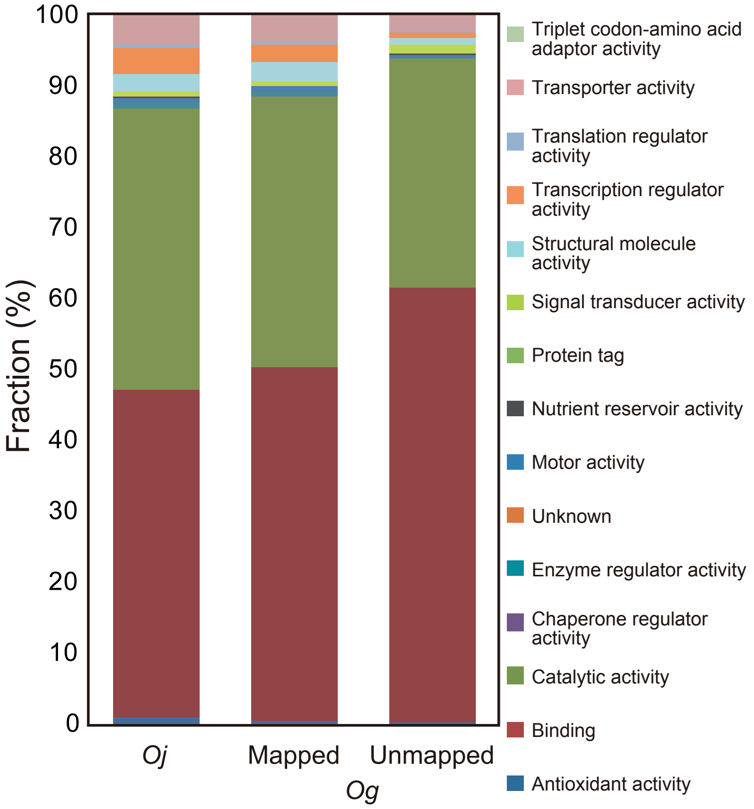

Supplement: Additional file 9 — Functional classifications of the proteins of Oj and two close relatives, On and Og. The classifications of mapped and unmapped BESs of On and Og were derived from the nr database proteins that were homologous to the mapped and unmapped BESs. Protein categories were based on the molecular functions of the Gene Ontology (GO) hierarchy. [file 1471-2164-11-121-S9.PDF]
