# Supplementary material for: Massive gene losses in Asian cultivated rice unveiled by comparative genome analysis
Source: BMC Genomics. 2010 Feb 19;11:121. doi: 10.1186/1471-2164-11-121 (PMC2831846; doi:10.1186/1471-2164-11-121)

Additional Data File 10. Functional classifications of the proteins of *Oj* and three close relatives, *On, Or*, and *Og*. Unlike in Additional Data File 9, all BESs were mapped to the genome of *O. sativa* L. ssp. *indica* (*Oi*). The classifications of mapped and unmapped BESs of the close relatives were derived from nr proteins that were homologous to the mapped and unmapped BESs (see Methods in the main text). Protein categories were based on the molecular functions of the Gene Ontology (GO) hierarchy.

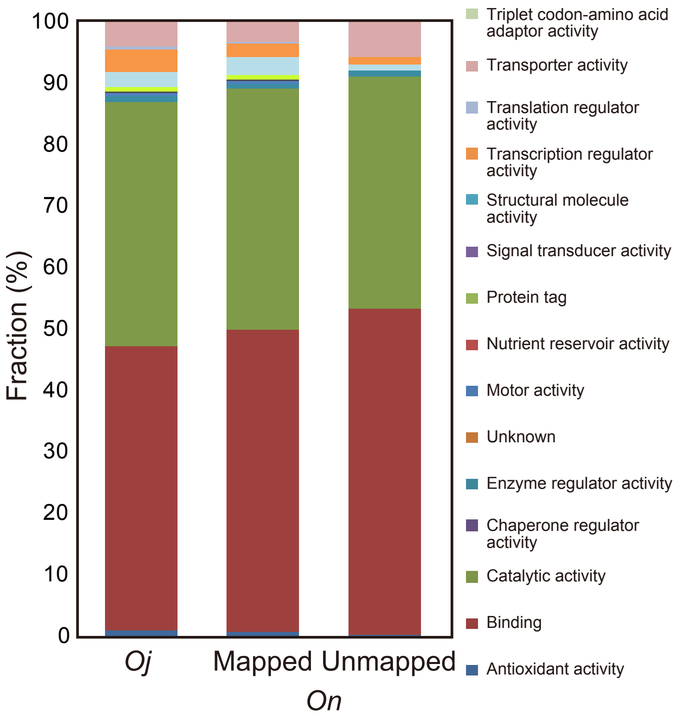

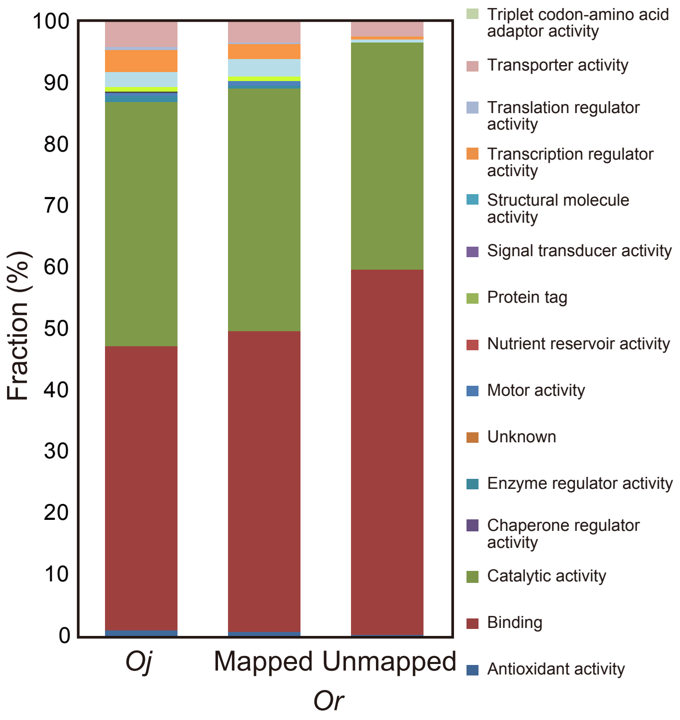

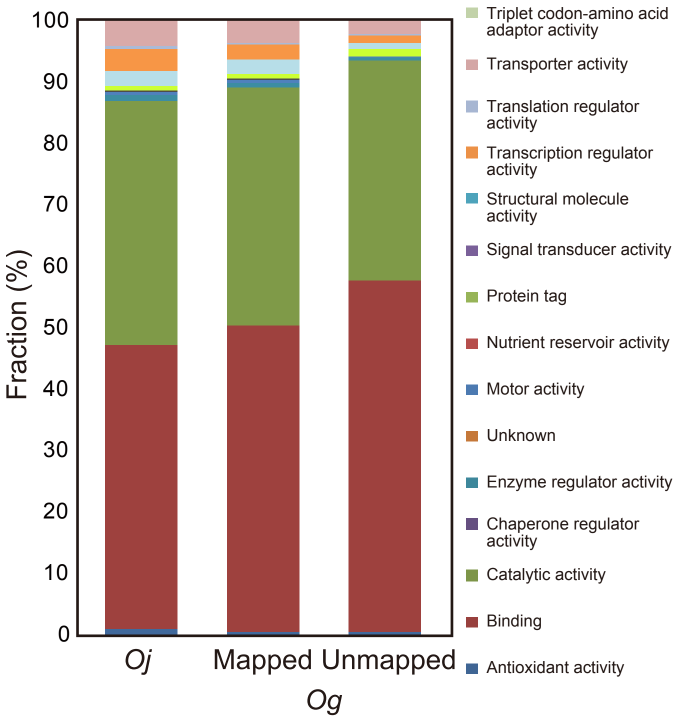

Supplement: Additional file 10 — Functional classifications of the proteins of Oj and three close relatives, On, Or, and Og. All BESs were mapped to the genome of O. sativa L. ssp. indica (Oi). The classifications of mapped and unmapped BESs of the close relatives were derived from the nr database proteins that were homologous to the mapped and unmapped BESs. Protein categories were based on the molecular functions of the Gene Ontology (GO) hierarchy. [file 1471-2164-11-121-S10.PDF]
