# Supplementary material for: Massive gene losses in Asian cultivated rice unveiled by comparative genome analysis
Source: BMC Genomics. 2010 Feb 19;11:121. doi: 10.1186/1471-2164-11-121 (PMC2831846; doi:10.1186/1471-2164-11-121)

Additional Data File 11. Functional classifications of the proteins of *Oj* and of simulated BESs of *Oj* and *Oi*. Simulated BESs of *Oj* were mapped to the genome of *Oi*, and vice versa. The classifications of mapped and unmapped BESs were derived from the nr database proteins that were homologous to the mapped and unmapped BESs (see Methods in the main text). Protein categories were based on the molecular functions of the GO hierarchy.

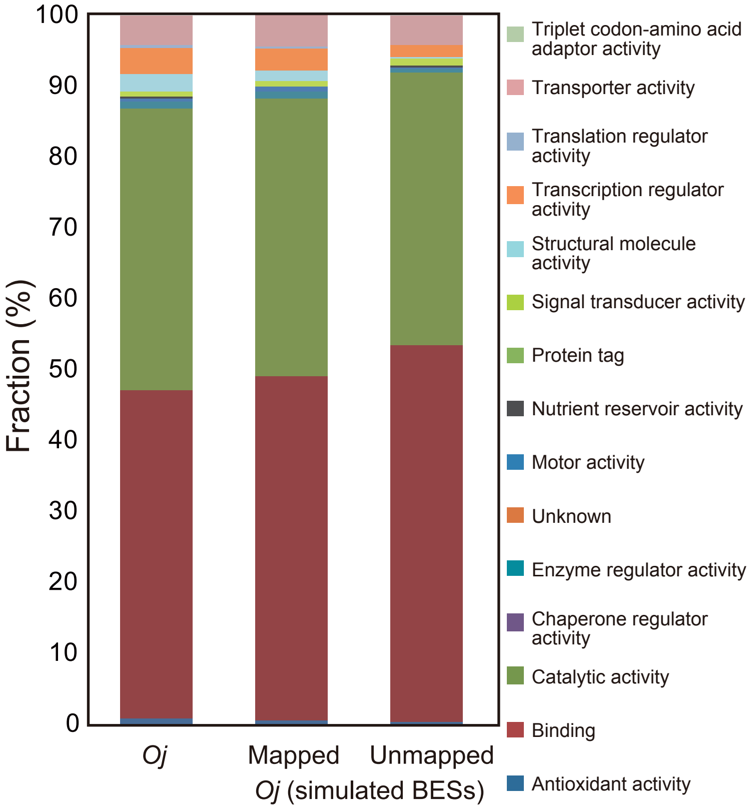

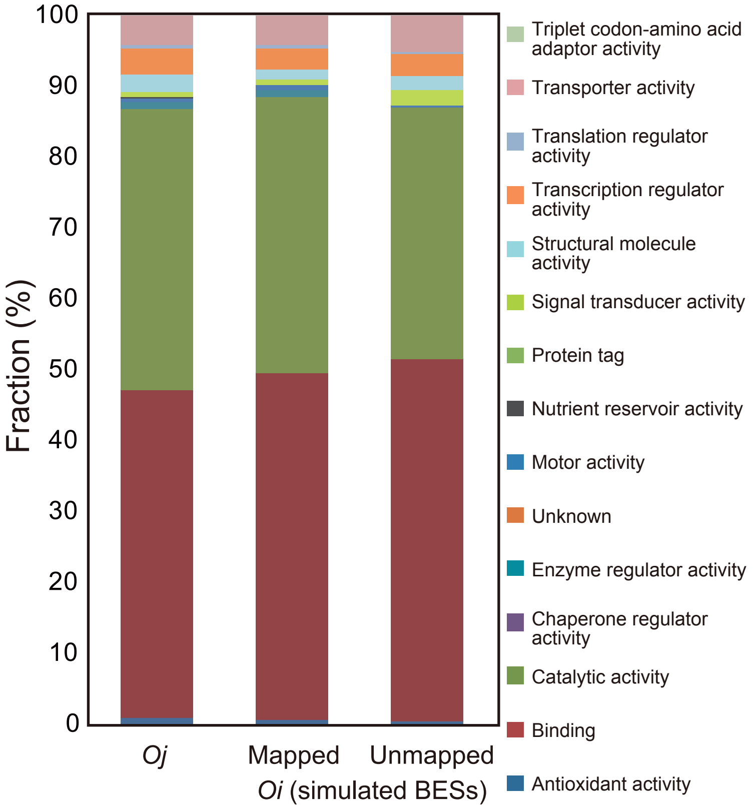

Supplement: Additional file 11 — Functional classifications of the proteins of Oj and of simulated BESs of Oj and Oi. Simulated BESs of Oj were mapped to the genome of Oi, and vice versa. The classifications of mapped and unmapped BESs were derived from nr database proteins that were homologous to the mapped and unmapped BESs. Protein categories were based on the molecular functions of the GO hierarchy. [file 1471-2164-11-121-S11.PDF]
