# Supplementary material for: Massive gene losses in Asian cultivated rice unveiled by comparative genome analysis
Source: BMC Genomics. 2010 Feb 19;11:121. doi: 10.1186/1471-2164-11-121 (PMC2831846; doi:10.1186/1471-2164-11-121)
Supplement: Additional file 12 — The ten most frequent domains among the unmapped BESs of On and Og. For each domain, the numbers of genes with or without the domain are listed for the mapped and unmapped BESs. P values were calculated using Fisher's exact test. [file 1471-2164-11-121-S12.PDF]

Additional Data File 12. The ten most frequent domains among the unmapped BESs of *On* and *Og*. For each domain, the numbers of genes with or without the domain are listed for the mapped and unmapped BESs. *P* values were calculated using Fisher's exact test.

O. nivara

InterPro ID	Description	Mapped		Unmapped		*p* value
		No. of genes having the domain	No. of genes without the domain	No. of genes having the domain	No. of genes without the domain	
IPR000719	Protein kinase, core	986	4696	41	190	0.86
IPR000767	Disease resistance protein	346	5336	39	192	2.12×10^{-8}
IPR001611	Leucine-rich repeat	715	4967	62	169	1.28×10^{-8}
IPR001878	Zinc finger, CCHC-type	176	5506	15	216	0.011
IPR002182	NB-ARC	399	5283	45	186	1.36×10^{-9}
IPR003593	AAA+ ATPase, core	203	5479	13	218	0.11
IPR008271	Serine/threonine protein kinase, active site	760	4922	31	200	1.52
IPR011009	Protein kinase-like	998	4684	43	188	0.66
IPR012337	Polynucleotidyl transferase, Ribonuclease H fold	116	5566	15	216	1.60×10^{-4}
IPR013210	Leucine-rich repeat, N-terminal	260	5422	28	203	5.83×10^{-6}

O. glaberrima

InterPro ID	Description	Mapped		Unmapped		*p* value
		No. of genes having the domain	No. of genes without the domain	No. of genes having the domain	No. of genes without the domain	
IPR000719	Protein kinase, core	775	3827	37	192	0.86
IPR000767	Disease resistance protein	300	4302	45	184	1.48×10^{-10}
IPR001611	Leucine-rich repeat	599	4003	66	163	8.72×10^{-10}
IPR001878	Zinc finger, CCHC-type	121	4481	19	210	2.65×10^{-5}
IPR002182	NB-ARC	341	4261	49	180	7.43×10^{-11}
IPR003593	AAA+ ATPase, core	185	4417	8	221	0.86
IPR008271	Serine/threonine protein kinase, active site	602	4000	29	200	0.92
IPR011009	Protein kinase-like	779	3823	36	193	0.72
IPR012337	Polynucleotidyl transferase, Ribonuclease H fold	85	4517	11	218	5.33×10^{-3}
IPR013210	Leucine-rich repeat, N-terminal	226	4376	24	205	1.03×10^{-3}
