# Supplementary material for: Massive gene losses in Asian cultivated rice unveiled by comparative genome analysis
Source: BMC Genomics. 2010 Feb 19;11:121. doi: 10.1186/1471-2164-11-121 (PMC2831846; doi:10.1186/1471-2164-11-121)
Supplement: Additional file 13 — List of 83 disease resistance-related genes that matched the unmapped BESs of On, Or, and Og. BESs that had internal stop codons within the aligned regions are presented in parentheses. [file 1471-2164-11-121-S13.PDF]

**Additional Data File 13.** List of 83 disease resistance-related genes that matched the unmapped BESs of *On*, *Or*, and *Og*. BESs that had internal stop codons within the aligned regions are presented in parentheses.

| Accession # | Definition | Accession # of BESs | | |
|---|---|---|---|---|
| | | *O. nivara* | *O. rufipogon* | *O. glaberrima* |
| AAX95851 | NB-ARC domain, putative [Oryza sativa (japonica cultivar-group)] | CL749184 | - | CW670370,(CW677751),CW765862 |
| ABG22484 | retrotransposon protein, putative, unclassified [Oryza sativa (japonica cultivar-group)] | - | (CL792545),(CL798160),(CL806925),(CL825974),(CL829057),(CL843851),(CL862603) | - |
| BAC45136 | putative nucleotide-binding leucine-rich-repeat protein 1 [Oryza sativa Japonica Group] | - | - | CW653072 |
| BAC84181 | putative stripe rust resistance protein Yr10 [Oryza sativa Japonica Group] | - | - | (CW668819),CW668896,CW684615 |
| BAD23621 | blight resistance protein SH20-like [Oryza sativa Japonica Group] | - | - | CW758101 |
| CAD45031 | NBS-LRR disease resistance protein homologue [Hordeum vulgare] | - | CL837032 | - |
| CAH66367 | OSIGBa0130K07.3 [Oryza sativa (indica cultivar-group)] | - | CL823783,CL839857,CL842602 | - |

| | | | | |
|---|---|---|---|---|
| CAJ86357 | OSIGBa0117N13.1 [Oryza sativa (indica cultivar-group)] | - | CL805480,CL846233 | - |
| EAY74323 | hypothetical protein OsI_002170 [Oryza sativa (indica cultivar-group)] | CL782225 | - | - |
| EAY77774 | hypothetical protein OsI_031733 [Oryza sativa (indica cultivar-group)] | (CL707703),CL738086 | - | - |
| EAY77791 | hypothetical protein OsI_031750 [Oryza sativa (indica cultivar-group)] | - | (CL811599) | CW672293 |
| EAY78567 | hypothetical protein OsI_032526 [Oryza sativa (indica cultivar-group)] | - | - | CW661847,CW852671, CW852932 |
| EAY80341 | hypothetical protein OsI_034300 [Oryza sativa (indica cultivar-group)] | CL762759 | - | (CW666128) |
| EAY80342 | hypothetical protein OsI_034301 [Oryza sativa (indica cultivar-group)] | - | CL837148 | - |
| EAY80354 | hypothetical protein OsI_034313 [Oryza sativa (indica cultivar-group)] | CL611592,CL614106,CL712791,CL716481,CL717111,CL722882,CL724159,CL741613,CL746743 | - | - |
| EAY80850 | hypothetical protein OsI_034809 [Oryza sativa (indica cultivar-group)] | CL614473,CL748892 | - | - |
| EAY80990 | hypothetical protein OsI_034949 [Oryza sativa (indica cultivar-group)] | - | CL795284,CL824418 | - |

| | | | | |
|---|---|---|---|---|
| EAY80991 | hypothetical protein OsI_034950 [Oryza sativa (indica cultivar-group)] | - | - | CW852758 |
| EAY81273 | hypothetical protein OsI_035232 [Oryza sativa (indica cultivar-group)] | CL776180 | - | (CW654326),CW670822 |
| EAY81275 | hypothetical protein OsI_035234 [Oryza sativa (indica cultivar-group)] | CL774927 | - | - |
| EAY81279 | hypothetical protein OsI_035238 [Oryza sativa (indica cultivar-group)] | CL783938 | - | CW756027 |
| EAY81540 | hypothetical protein OsI_035499 [Oryza sativa (indica cultivar-group)] | CL618156,CL712212,CL712302,(CL712765),CL718478,CL775801,CL779806 | - | - |
| EAY81542 | hypothetical protein OsI_035501 [Oryza sativa (indica cultivar-group)] | CL613014 | - | - |
| EAY81558 | hypothetical protein OsI_035517 [Oryza sativa (indica cultivar-group)] | - | - | CW767317 |
| EAY81588 | hypothetical protein OsI_035547 [Oryza sativa (indica cultivar-group)] | CL776767,CL783354,CL786904 | - | CW659764,CW660194,CW678779,(CW680210),CW683836 |
| EAY81809 | hypothetical protein OsI_035768 [Oryza sativa (indica cultivar-group)] | CL610961,CL628776,CL721164,CL731498,CL750448,CL754979,CL758657,CL759929,CL781294 | - | CW665546,CW757376 |

| | | | | |
|---|---|---|---|---|
| EAY82533 | hypothetical protein OsI_036492 [Oryza sativa (indica cultivar-group)] | CL783020 | - | - |
| EAY83109 | hypothetical protein OsI_037068 [Oryza sativa (indica cultivar-group)] | CL614928 | - | - |
| EAY83190 | hypothetical protein OsI_037149 [Oryza sativa (indica cultivar-group)] | CL721594 | CL814175,CL860616 | CW657851,CW680471, CW685182 |
| EAY83206 | hypothetical protein OsI_037165 [Oryza sativa (indica cultivar-group)] | CL760319 | - | CW679727 |
| EAY83316 | hypothetical protein OsI_037275 [Oryza sativa (indica cultivar-group)] | CL613148,CL617673,CL754391,CL757080,CL773091,(CL787016) | - | - |
| EAY83535 | hypothetical protein OsI_037494 [Oryza sativa (indica cultivar-group)] | CL776328 | CL796834,CL832417,CL841436 | - |
| EAY83683 | hypothetical protein OsI_037642 [Oryza sativa (indica cultivar-group)] | - | - | CW685144 |
| EAY83937 | hypothetical protein OsI_037896 [Oryza sativa (indica cultivar-group)] | - | CL815971,CL822315 | - |
| EAY85750 | hypothetical protein OsI_006983 [Oryza sativa (indica cultivar-group)] | - | - | (CW679060),CW770578 |
| EAY85833 | hypothetical protein OsI_007066 [Oryza sativa (indica cultivar-group)] | - | - | CW759030 |
| EAY85953 | hypothetical protein OsI_007186 [Oryza sativa (indica cultivar-group)] | - | - | CW761840,CW775279 |

| | | | | |
|---|---|---|---|---|
| EAY88487 | hypothetical protein OsI_009720 [Oryza sativa (indica cultivar-group)] | - | - | CW659494,CW686722 |
| EAY94423 | hypothetical protein OsI_015656 [Oryza sativa (indica cultivar-group)] | CL743712,CL783159 | CL793303,CL814927,CL820754,CL844174 | CW657010,CW685554, CW685973,CW774749, CW852677 |
| EAY96767 | hypothetical protein OsI_018000 [Oryza sativa (indica cultivar-group)] | - | - | CW667981 |
| EAY97677 | hypothetical protein OsI_018910 [Oryza sativa (indica cultivar-group)] | CL622853,CL748131 | - | - |
| EAY99388 | hypothetical protein OsI_020621 [Oryza sativa (indica cultivar-group)] | CL751549 | - | CW669415,CW697744 |
| EAZ02190 | hypothetical protein OsI_023422 [Oryza sativa (indica cultivar-group)] | CL630273,CL759569 | - | - |
| EAZ03120 | hypothetical protein OsI_024352 [Oryza sativa (indica cultivar-group)] | CL765834 | - | CW658575,(CW681989) |
| EAZ03122 | hypothetical protein OsI_024354 [Oryza sativa (indica cultivar-group)] | - | - | (CW664493),CW759368 |
| EAZ03692 | hypothetical protein OsI_024924 [Oryza sativa (indica cultivar-group)] | CL713592 | CL814662,CL862541 | - |
| EAZ03693 | hypothetical protein OsI_024925 [Oryza sativa (indica cultivar-group)] | CL611025,CL620245,CL743404,CL756501,CL756700,CL778428,CL779246 | CL819114,CL844980,CL858131,CL861305 | CW771577 |

| | | | | |
|---|---|---|---|---|
| EAZ03865 | hypothetical protein OsI_025097 [Oryza sativa (indica cultivar-group)] | - | CL798530,CL819115 | - |
| EAZ06155 | hypothetical protein OsI_027387 [Oryza sativa (indica cultivar-group)] | - | CL798426,(CL818660) | (CW757530) |
| EAZ06180 | hypothetical protein OsI_027412 [Oryza sativa (indica cultivar-group)] | - | - | CW665209,CW754559 |
| EAZ06896 | hypothetical protein OsI_028128 [Oryza sativa (indica cultivar-group)] | CL750077 | - | - |
| EAZ07816 | hypothetical protein OsI_029048 [Oryza sativa (indica cultivar-group)] | - | CL813643,CL819418 | CW681957 |
| EAZ08545 | hypothetical protein OsI_029777 [Oryza sativa (indica cultivar-group)] | - | - | CW656704 |
| EAZ08556 | hypothetical protein OsI_029788 [Oryza sativa (indica cultivar-group)] | - | CL822247 | - |
| EAZ08771 | hypothetical protein OsI_030003 [Oryza sativa (indica cultivar-group)] | CL720299,CL726851 | - | - |
| EAZ08819 | hypothetical protein OsI_030051 [Oryza sativa (indica cultivar-group)] | - | - | CW693069 |
| EAZ09172 | hypothetical protein OsI_030404 [Oryza sativa (indica cultivar-group)] | (CL624144),CL721448, CL747839,CL765220 | CL810581 | - |
| EAZ11520 | hypothetical protein OsJ_001345 [Oryza sativa (japonica cultivar-group)] | CL710360,CL755881,CL787021 | CL821666 | - |

| | | | | |
|---|---|---|---|---|
| EAZ17814 | hypothetical protein OsJ_032023 [Oryza sativa (japonica cultivar-group)] | CL612832,CL707682 | CL823776,CL861588 | - |
| EAZ17854 | hypothetical protein OsJ_032063 [Oryza sativa (japonica cultivar-group)] | - | CL841334 | - |
| EAZ17877 | hypothetical protein OsJ_032086 [Oryza sativa (japonica cultivar-group)] | - | CL803429 | - |
| EAZ18355 | hypothetical protein OsJ_032564 [Oryza sativa (japonica cultivar-group)] | - | - | CW663785,CW665381, CW669074,CW691226, CW755165,(CW773029) |
| EAZ18403 | hypothetical protein OsJ_032612 [Oryza sativa (japonica cultivar-group)] | - | CL805020 | CW666918 |
| EAZ18867 | hypothetical protein OsJ_033076 [Oryza sativa (japonica cultivar-group)] | - | (CL800266),(CL852113) | - |
| EAZ19037 | hypothetical protein OsJ_033246 [Oryza sativa (japonica cultivar-group)] | CL767306 | - | - |
| EAZ19226 | hypothetical protein OsJ_033435 [Oryza sativa (japonica | CL746564,CL758336,CL 763990 | - | CW754518,CW757687 |

| | | | | |
|---|---|---|---|---|
| | cultivar-group)] | | | |
| EAZ39030 | hypothetical protein OsJ_022513 [Oryza sativa (japonica cultivar-group)] | - | - | (CW653528),(CW689977),(CW764637) |
| EAZ39807 | hypothetical protein OsJ_023290 [Oryza sativa (japonica cultivar-group)] | - | CL815333,CL833700,CL858060,CL862487 | CW687805,CW759552 |
| EAZ40863 | hypothetical protein OsJ_024346 [Oryza sativa (japonica cultivar-group)] | CL709211,CL717799,CL743491 | - | CW663956 |
| EAZ41791 | hypothetical protein OsJ_025274 [Oryza sativa (japonica cultivar-group)] | - | - | (CW660766) |
| EAZ44276 | hypothetical protein OsJ_027759 [Oryza sativa (japonica cultivar-group)] | - | CL797047 | CW773512 |
| EAZ44473 | hypothetical protein OsJ_027956 [Oryza sativa (japonica cultivar-group)] | - | CL824914 | (CW662024) |
| EAZ44474 | hypothetical protein OsJ_027957 [Oryza sativa (japonica cultivar-group)] | - | - | CW657610,CW755985, CW772711 |

| | | | | |
|---|---|---|---|---|
| NP_001057389 | Disease resistance protein family protein. | CL625725 | - | CW760499 |
| NP_001059355 | Transcriptional factor B3 family protein. | CL775153 | - | - |
| NP_001062828 | Disease resistance protein family protein. | - | - | CW660160,CW686461, CW761535 |
| NP_001064207 | Disease resistance protein family protein. | (CL785928) | - | - |
| NP_001067488 | Similar to NBS-LRR disease resistance protein homologue (Fragment). | - | CL854607,CL862278 | - |
| NP_001067491 | Similar to NBS-LRR disease resistance protein homologue (Fragment). | - | CL796711,CL853123 | CW695546 |
| NP_001067529 | Disease resistance protein family protein. | CL714031 | - | - |
| NP_001068305 | Ras GTPase family protein. | - | CL797566 | - |
