# Supplementary material for: Massive gene losses in Asian cultivated rice unveiled by comparative genome analysis
Source: BMC Genomics. 2010 Feb 19;11:121. doi: 10.1186/1471-2164-11-121 (PMC2831846; doi:10.1186/1471-2164-11-121)
Supplement: Additional file 14 — Amino acid alignment between the On-specific disease resistance gene, CL716448, and its homologues. Amino acid sequences with black backgrounds indicate leucine-rich repeat domains predicted by InterProScan searches. [file 1471-2164-11-121-S14.PDF]

Additional Data File 14. Amino acid alignment between the *On*-specific disease resistance gene, CL716448, and its homologues. Amino acid sequences with black backgrounds indicate leucine-rich repeat domains predicted by InterProScan searches.

```
AAK20742 (LRR14) LQVVERNYAGRLKWCRMHDVIRLLALNKAKEECFGKVYNGSGGTRAFSVEGARRISVLGG
AAK20736 (LRR19) LQVVERNYAGRLKECRMHDVIRLLALNKAKEECFGNVYNGSGGTGVFSIEGARRISVLGG
AAD46469         LRVVQRNHTGRLKCCQMHDVIRLLALNKAKEEFFGKGYNGSGCTGAFSVEGARRISVQCG
CL716448         LQVVKRNVVGRVKCCRIHDIVRLLVLTKANEECFCQIYDVS---RAFSVEDTRRISIQSV
EAY94423         LQVVERNRNGEVNTCRMHDIIRILGLAKS-EECFCRVYDGS---RAFLAEGIRRLSIQSS
EAZ15395         LQVVERNESGRVRSCRMHDIIRILALTKSNEESFCSVYDGS---RTTSKQNTRRLSIQSS
                 *:**:**  *.:. *::**::*:* * *: ** *   *: *    .   :. **:*:

AAK20742 (LRR14) NIEQLSLSGTTQLRALHVFEKYINVDLLKPILTSSNLLSTLDLQGTRIKMLPDEVFDLFN
AAK20736 (LRR19) NIEQLSLSGATQLRALHVFESYIDIDLLKPILTSSSLLSTLDLQGTHIKMLPNEVFDLFN
AAD46469         NLEQLSRSCARHLRALHVFERYINVDLLKPIITSSNLLSTLDLQGTNIKMLPNEVFNLFN
CL716448         NIEQFIPSGTTRLRALYVFESNIKIIMLRPILKSSIFLSTLDLQGAQFKMLPPEVFNLFN
EAY94423         NVNQLSQSGAPHLRHLYIFGSGLSIDSLTPFLKSFSLLSSLDLQGVNMKSLPHVVFKLYN
EAZ15395         DIEKFTVSSEVHLRAIYAFNELVTSDSLKFFLKSFNLLSTLDLQGTQIRKLPKELFKLFN
                 :::::  *   :** :: *   :    *  ::.*  :**:*****..:: **  :*.*:*

AAK20742 (LRR14) LRYLGIRDTEIESLPEAVGRLQNLEVLDAFESKLMYLPNNVVKLRKLRYLYACPPST--S
AAK20736 (LRR19) LRYLGLRDTKIESMPAAVGRLQNLQVLDAYHSKLTYLPNSVVKLQKLRYLYAG------T
AAD46469         LRYLGLRNTEVEILPEVVGRLQNLEVLDALESKLMYLPKSIVKLRKLRYLYVCTPAT--S
CL716448         LRYLGLRHTHIEVLPEAIGRLRNLEVLDAVNTKLVYLPNNVVKLQKLRYLYACTFGD--G
EAY94423         LRFLGLRDTDIAIIPRSIGRLRHLEVLDARNTKLMTLPKDIVQLQKLRYLNVDTIPEEAD
EAZ15395         LHFLCLRDTFVEDIPETVGRLQKLEVLDAFNARLVSLPQSIANLHKLRYLYVATDPRKGT
                 *::* :*.* :  :*  :***::*:**** .::*  **:.:.:*:***** .

AAK20742 (LRR14) EAVN-VGGVKVPNGMQHLTGLQALQFVDASLEFLREVGDLTELRTFGVCNVRSEHSADL-
AAK20736 (LRR19) WKDS-IRGVKVPKGMQHLAGLHALQSVRATPEFLHEAAALTELRTFDVCNVQSEHSAYL-
AAD46469         ETVT-IGGVKVPNVMQHLAGLRALQSIKATPEFLREVAALTELRTFDVCDVRSEHSADL-
CL716448         KSVQPFGGVKLPIGIRYLTGLRALQCAKATSEALTEIGALTELRTFGVTDVKNKHSGSL-
EAY94423         RKVVFFGGIRVPTGIEQLTRLQTLQLVEASTETLCHIGSLTQLRAFAVNKVRNEHCVHLC
EAZ15395         KGVVPWIGIQVPNGIRNLKSLQALQLVEANSETLCHLGALTELRTFAITQVRREQCSDLC
                        *:::*  :. *  *::**   *. * * . . **:**:* : .*: ::.  *
```
