# Supplementary material for: Massive gene losses in Asian cultivated rice unveiled by comparative genome analysis
Source: BMC Genomics. 2010 Feb 19;11:121. doi: 10.1186/1471-2164-11-121 (PMC2831846; doi:10.1186/1471-2164-11-121)
Supplement: Additional file 15 — Phylogenetic tree of possible disease resistance proteins. CL619881 is a newly found homologue from On. Accession numbers and species names are shown. The tree was reconstructed by the neighbour-joining method. The interior branches were tested by 1,000 bootstrap replicates, and bootstrap values of 50% or greater are shown above the branches. The scale indicates the branch length. [file 1471-2164-11-121-S15.PDF]

Additional Data File 15. Phylogenetic tree and amino acids alignment of possible disease resistance proteins. CL619881 is a newly found homologue from *On.* Accession numbers and species names are shown. The tree was reconstructed by the neighbour-joining method. The interior branches were tested by 1000 bootstrap replicates, and bootstrap values of 50% or greater are shown above the branches. The scale indicates the branch length.

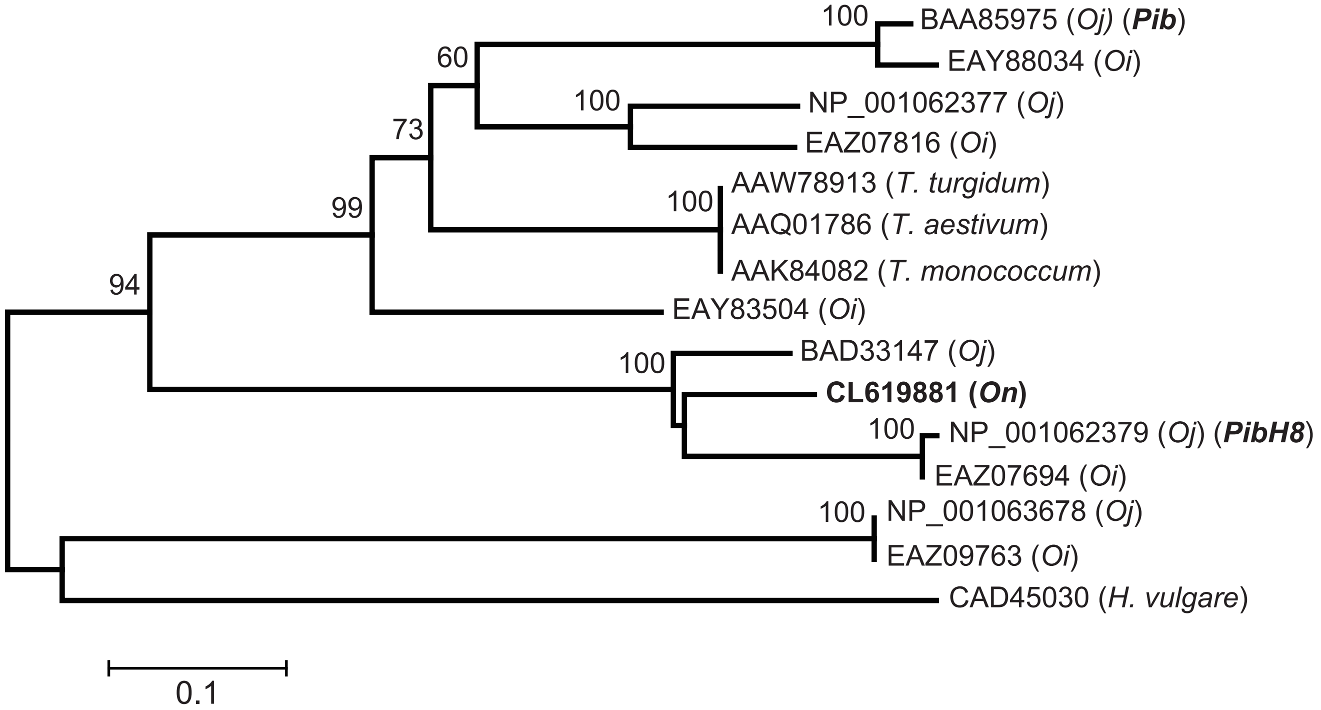


```
CL619881     AIAAVGGFLSTRPQNITEWRKFSDHIS-AELDNSXSLEMINKILISSYEGLSYHLKSCFL
BAD33147     AIVAVGGFLSTRPPNIREWRKFSDHIS-TELDENPSLEMIKKILISSYEGLSYHLKSCFL
NP_001062379 AVATVGGFLSARPRNIIEWREFSDRIS-EEFDNNPSLEMIKKILASSYEGLTYHLKSCFL
EAZ07694     AVATVGGFLSARPRNIIEWREFSDRIS-EEFDNNPSLEMIKKILASSYEGLTYHLKSCFL
BAA85975     AIVVIGGFLANRPKTPEEWRKLNENIN-AELEMNPELGMIRTVLEKSYDGLPYHLKSCFL
EAY88034     AIVVIGGFLANRPKTPEEWRKLNENIN-AELEMNPELGMIRTVLEKSYDGLPYHLKSCFL
NP_001062377 AIVTIGGFLANQPKTALAWRKINEHIS-AELEMNPELKTISTVLLKSYDGLPYKLKSCFL
EAZ07816     AIVTIGGFLASQPKSALAWRKLNEHIN-AELEMNPKLETIKTVLLKSYDGLPFHLKSCFL
AAQ01786     AIVTIGGFLANQPKTALEWKKLNEHISAAELQMNPELEAIITVLNKSYDGLPYHLKSCFL
AAK84082     AIVTIGGFLANQPKTALEWKKLNEHISAAELQMNPELEAIITVLNKSYDGLPYHLKSCFL
AAW78913     AIVTIGGFLANQPKTALEWKKLNEHISAAELQMNPELEAIITVLNKSYDGLPYHLKSCFL
EAY83504     AIVTIGGFLASSPKTALEWRKLNEHIS-AELETNPGLEAIRAVLNISYDGLPYHLKSCFL
CAD45030     AVLTIGGMLAN--KKVTEWESIYKQIP-SELESNPSLEAMRRIVTLSYNNLPSHLKSCFL
EAZ09763     AIATIGGLLANRPKTSAEWKNLRIHLG-SELEFDQDINSINRVITSSYDGLPYHLKSCFL
NP_001063678 AIATIGGLLANRPKTSAEWKNLRIHLG-SELEFDQDINSINRVITSSYDGLPYHLKSCFL
             *: .:**:*:    .   *..:  .:   *:: .  :  :  ::  **:.*. :******
```

```
CL619881     NSGKTGFCQLHDLMREISVSKSEEENLVLVLDDHSTSRSKDKVRHLVVSQT---WSR---
BAD33147     -TGKTGFCQVHDLMREISIAKSEEENLVLVLDEHSISSSKDKVRHLVISQS---WSREQ-
NP_001062379 -SGKTGFCQVYNLMHEIIISKSEEENLVLVLDDHITSRSKDKVRHLVVSKS---WSR---
EAZ07694     -SGKTGFCQVYDLMHEIIISKSEEENLVLVLDDHITSRSKDKVRHLVVSKS---WSR---
BAA85975     SRKSIDSCKVHDLMRDIAISKSTEENLVFRVEEGCSAYIHGAIRHLAISSN---WKG---
EAY88034     SRKSIDSCKVHDLIRDIAISKSTEENLVFRMEEGCSAYIHGAIRHLAISSN---WKG---
NP_001062377 PEGGIDYCQLHDLMREISITKSMEENLVLRLEKGCSSKTQGTARHLAISSN---WEG---
EAZ07816     VKQGIDYCQLHDLMREISITKSMEENLVFRLEEGCSSNNQGTTRHLAISSN---WEG---
AAQ01786     DGKRIGSCQVHDLIREIGISKSMEGNLVLRLEEGCSLNTQGTARHLAISSN---WER---
AAK84082     DGKRIGSCQVHDLIREIGISKSMEGNLVLRLEEGCSLNTQGTARHLAISSN---WER---
AAW78913     DGKRIGSCQVHDLIREIGISKSMEGNLVLRLEEGCSLNTQGTARHLAISSN---WER---
EAY83504     YNNRPDSCQVHDIMREIAISKSKEENLVLRLEGGHRLHNHGIVRHLSITNSGEDRET---
CAD45030     IEGHVKSCRVHDIMRDVMVSISREENFVYLAGDNSTRVAEGNFRHVAYHGS---------
EAZ09763     ASMAVERCQVHGMVLQIILSKSIEENQLFIIDKHCNEVPQSKIRHLVVTR----WKR---
NP_001063678 ASMAVERCQVHGMVLQIILSKSIEENQLFIIDKHCNEVPQSKIRHLVVTR----WKR---
                     *:::.:: :: :: * * * :           ..  **:
```

```
CL619881     YLSIFPEDHNIRHGRLLRRWIAEGYSRATRNKNAEKEAEEQFMILLNKSMIQQ-SRTITS
BAD33147     YLSIFPEDHDIRYGRLLRRWIAEGYSRAKRNNNAEKEAEEQFTALLNKSMIQQ-SRTVT-
NP_001062379 YMSIFPEDSDIRYRRLLRRWTAEGYSRATRNRSNEKVAEEQFTALLNKSMIQQ-SKTIA-
EAZ07694     YMSIFPEDSDIRYRRLLRRWTAEGYSRATRNRSNEKVAEEQFTALLNKSMIQQ-SKTIA-
BAA85975     YLSIFPEDQIISRRRLVHRWAAEGYSTAAHGKSAIEIANGYFMELKNRSMILP-FQQSGS
EAY88034     YLSIFPEDQIISRRRLVHRWAAEGYSTAAHGKSAIEIANGYFMELKNRSMILP-SQQSAH
NP_001062377 YLSIFPEDHKVSRKRLMQRWTAEGYTTEARGKSPIEIAHDNFIELISRSMTLPVAQNASR
EAZ07816     YLSIFPEDYKVNRKRLMQRWTAEGYTVEMRGKPKKEIAHDIFMELISRSMILP-AQESIE
AAQ01786     YLSIFPEDYNIKLKRLLRRWIAEGYPGVVRNKSTEEVAESYFMDLISRSMLLP-SQRSIC
AAK84082     YLSIFPEDYNIKLKRLLRRWIAEGYPGVVRNKSTEEVAESYFMDLISRSMLLP-SQRSIC
AAW78913     YLSIFPEDYNIKLKRLLRRWIAEGYPGVVRNKSTEEVAESYFMDLISRSMLLP-SQRSIC
EAY83504     YLSIFPEDDKISRERLVRRWCAEGYSRELWDKSAEEIANNYFFELIDRSMILP--TQKLT
CAD45030     YLSIFPEDFEIKRRRLVDRWIAEGFVRARSGVNIEDVGISYFTELINRSMIQP---SKVS
EAZ09763     YLSIFPENHEIRYTRLVRRWIAEGYIAKRRDMTVEEVGQKHYNDLMNRSMIRP-MKKKIG
NP_001063678 YLSIFPENHEIRYTRLVRRWIAEGYIAKRRDMTVEEVGQKHYNDLMNRSMIRP-MKKKIG
             *:*****:  :   **: ** ***:      .    . .    :  * .:**
```

```
CL619881     ---KKKNDMQNIVDVSHIRSLTVFG----EWKSFFISKKMRMLRVLALEDARGLQDHDLVPVG
BAD33147     ---KKNNDMQNIVDVSHIRSLTVFG----EWKSFFLSKKMRMLRVLDLEDTDGLEDHDIVPIG
NP_001062379 ----EKNDMQNIVDVSHIRSLTVFG----EWKSFFLSKKMRMLRVLDLEDAEGLQDPDLVPIG
EAZ07694     ----EKNDMQNIVDVSHIRSLTVFG----EWKSFFLSKKMRMLRVLDLEDAEGLQDPDLVPIG
BAA85975     ----DKSEFEGIVDLSRIRSLSLFG----DWKPFFVYGKMRFIRVLDFEGTRGLEYHHL----
EAY88034     ----DKSEFEGIVDLSQIRSLSLFG----HWEPFFVSSKMKFIRVLDFERTRGLKYHHL----
NP_001062377 ----DKHEFENTVDLSRVRSLTVFG----KWKPFFVSEKMRMLRVLDLEDTEGLVDHHLEHIG
EAZ07816     ----DKHEFESMVDLSRLRSLTVFG----EWKPFFISKKMRMLRVLDLEDTDNLVDHHLEHIG
AAQ01786     ----DQSAFESIVDMSRVRSITVFG----EWKPFFLSDKMRLLRVLDLEDTTGLVNHHLEHIG
AAK84082     ----DQSAFESIVDMSRVRSITVFG----EWKPFFLSDKMRLLRVLDLEDTTGLVNHHLEHIG
AAW78913     ----DQSAFESIVDMSRVRSITVFG----EWKPFFLSDKMRLLRVLDLEDTTGLVNHHLEHIG
EAY83504     ----DFAELETTVDMSRIRSLTVFG----EWKPFFISDKMRLLRVLDLEDTEGVRNHHIKQIG
CAD45030     ----KSQKIG--VDYSHVRSLTMFGERSMELSTSLFSSKLRMLRALDLAD-------------
EAZ09763     ----SEEKMATNINLSLVRSLTVFG----ECPASLISPKLRLLRVLDLENAVDLENDDLKHIG
NP_001063678 ----SEEKMATNINLSLVRSLTVFG----ECPASLISPKLRLLRVLDLENAVDLENDDLKHIG
                 .   :   :: * :**:::**    .  . :.  *::::*.* :
```
