# Supplementary material for: Massive gene losses in Asian cultivated rice unveiled by comparative genome analysis
Source: BMC Genomics. 2010 Feb 19;11:121. doi: 10.1186/1471-2164-11-121 (PMC2831846; doi:10.1186/1471-2164-11-121)

Additional Data File 16. Density of the BESs throughout the *Oj* genome. Numbers of the BESs were counted by using a sliding window of 1 Mbp width with step size of 500 Kbp.

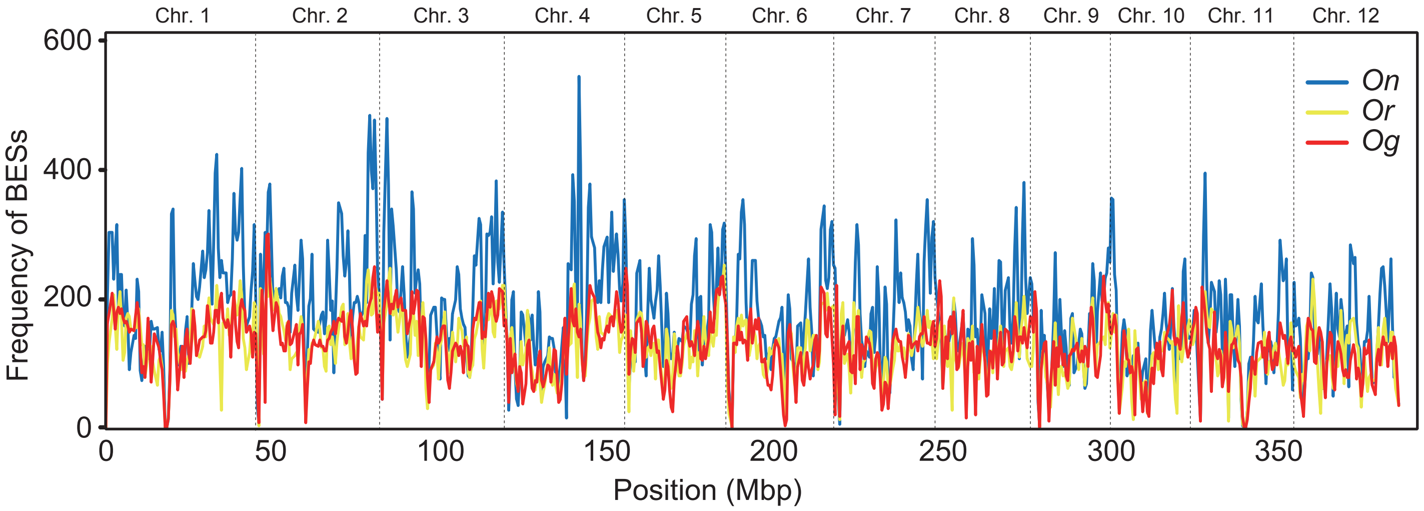

Supplement: Additional file 16 — Density of the BESs throughout the Oj genome. Numbers of the BESs were counted by using a sliding window of 1 Mbp width with a step size of 500 Kbp. [file 1471-2164-11-121-S16.PDF]
