# Supplementary material for: Massive gene losses in Asian cultivated rice unveiled by comparative genome analysis
Source: BMC Genomics. 2010 Feb 19;11:121. doi: 10.1186/1471-2164-11-121 (PMC2831846; doi:10.1186/1471-2164-11-121)

Additional Data File 18. Distributions of the amino acid identities of mapped BESs against the top-hit nr proteins with a threshold of 10^{-10}.

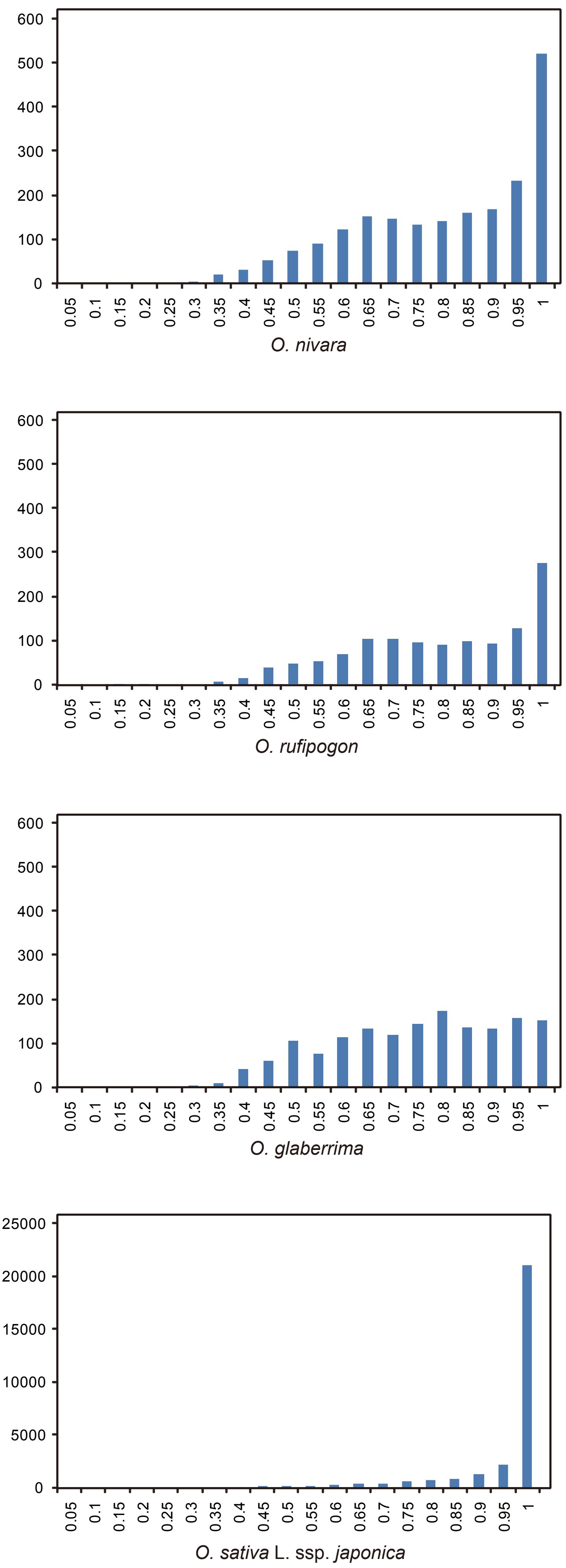

Supplement: Additional file 18 — Distributions of the amino acid identities of mapped BESs against the top-hit nr database proteins with a threshold of 10-10 [file 1471-2164-11-121-S18.PDF]
