# Supplementary material for: Massive gene losses in Asian cultivated rice unveiled by comparative genome analysis
Source: BMC Genomics. 2010 Feb 19;11:121. doi: 10.1186/1471-2164-11-121 (PMC2831846; doi:10.1186/1471-2164-11-121)

Additional Data File 19. Indirect functional classifications of the genes of *Oj*. We obtained protein-coding nucleotide sequences of representative sequences for each gene of *Oj* from RAP-DB and conducted BLASTX searches against the nr database with a threshold of 10^{-10}. Indirect classifications were facilitated using four sets of nr proteins: Top-hit, top-hit genes; Id=90%, nr proteins with amino acid identities over 90%; ID=80%, nr proteins with amino acid identities over 80%; ID=70%, nr proteins with amino acid identities over 70%. As a comparison, the functional classification of the representative sequences of the *Oj* genes (Rep) is shown on the right.

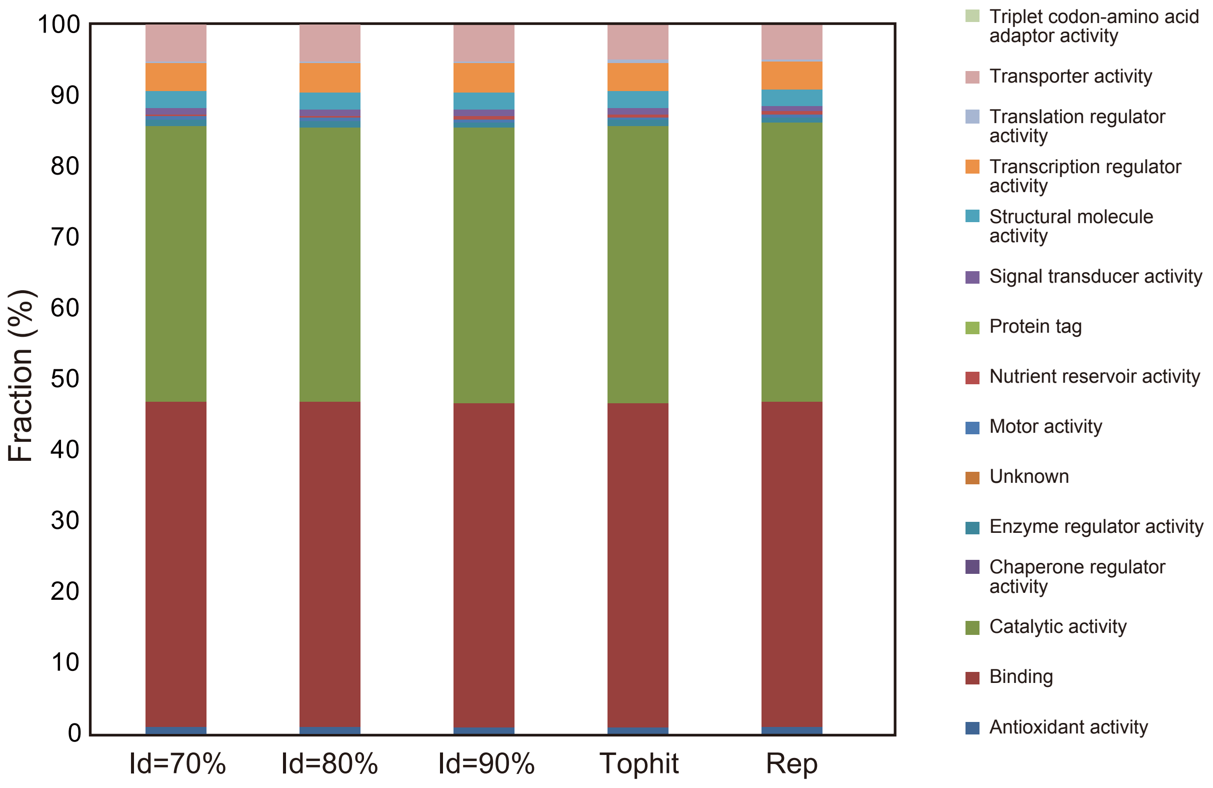

Supplement: Additional file 19 — Indirect functional classifications of the genes of Oj. We obtained protein-coding nucleotide sequences of representative sequences for each gene of Oj from RAP-DB and conducted BLASTX searches against the nr database with a threshold of 10-10. Indirect classifications were facilitated using four sets of the nr database proteins: Top-hit, top-hit genes; ID = 90%, nr database proteins with amino acid identities over 90%; ID = 80%, nr database proteins with amino acid identities over 80%; ID = 70%, nr database proteins with amino acid identities over 70%. As a comparison, the functional classification of the representative sequences of the Oj genes (Rep) is shown on the right. [file 1471-2164-11-121-S19.PDF]
